# A Novel Multi-Omics Analysis Model for Diagnosis and Survival Prediction of Lower-Grade Glioma Patients

**DOI:** 10.3389/fonc.2022.729002

**Published:** 2022-05-12

**Authors:** Wei Wu, Yichang Wang, Jianyang Xiang, Xiaodong Li, Alafate Wahafu, Xiao Yu, Xiaobin Bai, Ge Yan, Chunbao Wang, Ning Wang, Changwang Du, Wanfu Xie, Maode Wang, Jia Wang

**Affiliations:** ^1^ Department of Neurosurgery, The First Affiliated Hospital of Xi’an Jiaotong University, Xi’an, China; ^2^ Center of Brain Science, The First Affiliated Hospital of Xi’an Jiaotong University, Xi’an, China; ^3^ Department of Medical Imaging, The First Affiliated Hospital of Xi’an Jiaotong University, Xi’an, China; ^4^ Department of Pathology, The First Affiliated Hospital of Xi’an Jiaotong University, Xi’an, China

**Keywords:** LGG, multi-omics analysis, nomogram, radiomics, prediction

## Abstract

**Background:**

Lower-grade gliomas (LGGs) are characterized by remarkable genetic heterogeneity and different clinical outcomes. Classification of LGGs is improved by the development of molecular stratification markers including IDH mutation and 1p/19q chromosomal integrity, which are used as a hallmark of survival and therapy sensitivity of LGG patients. However, the reproducibility and sensitivity of the current classification remain ambiguous. This study aimed to construct more accurate risk-stratification approaches.

**Methods:**

According to bioinformatics, the sequencing profiles of methylation and transcription and imaging data derived from LGG patients were analyzed and developed predictable risk score and radiomics score. Moreover, the performance of predictable models was further validated.

**Results:**

In this study, we determined a cluster of 6 genes that were correlated with IDH mutation/1p19q co-deletion status. Risk score model was calculated based on 6 genes and showed gratifying sensitivity and specificity for survival prediction and therapy response of LGG patients. Furthermore, a radiomics risk score model was established to noninvasively assist judgment of risk score in pre-surgery. Taken together, a predictable nomogram that combined transcriptional signatures and clinical characteristics was established and validated to be preferable to the histopathological classification. Our novel multi-omics nomograms showed a satisfying performance. To establish a user-friendly application, the nomogram was further developed into a web-based platform: https://drw576223193.shinyapps.io/Nomo/, which could be used as a supporting method in addition to the current histopathological-based classification of gliomas.

**Conclusions:**

Our novel multi-omics nomograms showed the satisfying performance of LGG patients and assisted clinicians to draw up individualized clinical management.

## Introduction

Gliomas are infiltrative neoplasms of the central nervous system that exhibit variable genetic heterogeneity, epigenetic signatures, and clinical outcomes (1). According to histopathologic characteristics and morphologic signatures, gliomas are divided into 4 subgroups (2). The lower-grade glioma (LGG) is defined as pathological grade I to grade III glioma, which is consistent with the genetic categorization from The Cancer Genome Atlas (TCGA) (3). Epidemiologically, LGGs account for approximately 20% of all gliomas and present a significant indolent course with most lethal morbidity among patients from 35 to 45 years old (4, 5). The prognosis and therapeutic sensitivity of LGGs vary remarkably due to the different gene signatures (6). After receiving effective therapies, most LGG patients exhibit more favorable prognosis. However, a smaller subset of infiltrative LGGs shows more significant invasion and rapid progression to glioblastoma (grade IV glioma) even after receiving maximum treatment (7). Traditional classification strategies have barely reflected the heterogeneity of LGGs. Therefore, development of more precise and reliable identification of LGGs is essential for individual precision treatment of LGG patients.

Studies based on multi-omics analysis have corroborated a wide range of molecular biomarkers that are crucial to glioma subtype identification, prognosis prediction, and individualized therapy selection of LGG (2). Accumulating data have indicated that the mutation status of isocitrate dehydrogenase (IDH) and the integrity status of chromosome 1p and 19q (1p/19q) provide superior prognostic implication in comparison to the classical histopathological classification of LGGs (8). Comprehensive transcriptional analysis using TCGA Research Network indicates that IDH mutation and 1p/19q combined deletion (IDH^mut^/1p19q^codel^) gliomas reveal more favorable outcomes with a median overall survival (OS) of approximate 10 years. In contrast, IDH wild type and intact 1p/19q (IDH^wt^/1p19q^non-codel^) show more severe outcomes with a median survival period of 1.7 years (8). Therefore, evaluation of the IDH and 1p19q status has become a standard practice in the diagnosis of LGGs. However, recent studies have shown that the classification method based on IDH and 1p19q is deficient for stratification of risk for glioma patients (9). Chan et al. (10) find that IDH-mutated LGGs are not a homogeneous subtype as was originally thought; only 49 samples present longer progression-free survival and OS among 157 IDH-mutated LGGs. Similarly, the dramatically different survival was observed among patients with the same 1p19q status (11). Therefore, additional prediction biomarkers should be identified to establish more accurate management of LGG patients.

Accumulating evidence has reported the underlying molecular mechanism of malignant subtype transition, and radio-resistance and chemo-resistance of gliomas contradict the transcriptional aberrations and are correlated to DNA methylation alterations (12). Moreover, the aberrance of DNA methylation in the promoter regions of tumors is generally considered as a hallmark that contributes to the transcriptional downregulation of tumor suppressor genes and the upregulation of oncogenes (13). Binder et al. (14) report an integrative, multidimensional stratification of LGGs through a combination of genomic, epigenomic, and transcriptomic signatures to formulate individualization of treatment. Similarly, Mazor et al. (15) reveal that extensive interaction between genetics and epigenetics exists during the neoplasia of glioma, indicating that the reliable biomarkers should be identified through the combination of methylation and expression analysis. Nevertheless, only a portion of DNA methylation alterations generates malignant initiation or progression in tumor, which is similar to driver mutations that provide selective growth dominance and promote tumorigenesis (16). Therefore, identification of tumor progression-related types of DNA methylation alterations provides significant benefits to clarify the biological behavior and explore potential therapeutic targets of glioma. Bai et al. (17) find that the DNA methylation-driven gene (DMDG) signature is significantly associated with the OS of gastric cancer patients. Long et al. (18) also identify and validate two DMDGs with an advantageous accuracy for distinguishing hepatocellular carcinoma from normal samples and dysplastic nodules. However, the DMDGs that could be used for survival prediction and clinical management of LGG patients remain unknown.

Although the molecular biomarkers presented satisfying guidelines for patients, they also have a common deficiency due to the fact that the necessary information can only be obtained after surgical resection. Therefore, none of these biomarkers can be used for pre-surgical evaluation and management (19, 20). Magnetic resonance imaging (MRI) is a widely used noninvasive preoperative test that provides preliminary information regarding subtype and malignancy of brain tumors (21). It has been reported that conventional MRI features, including unilateral growth, sharpness of tumor margin, and heterogeneous intensity, are strongly relevant to prognosis. However, these features lack satisfactory precision and are dependent on radiologists’ subjective judgment and personal experience (22). Radiomics is an emerging research method based on MRI and has attracted substantial attention since it has the potential to provide spatial and temporal heterogeneity and present the accuracy of molecular marker predictions in glioma (23). Su et al. (24) demonstrate radiomics features that provide high discriminatory accuracy in predicting the H3 K27M mutation status of midline glioma; the aera under the curve (AUC) is 0.903. Therefore, radiomics analysis could provide a more elaborate investigation of multiple imaging features and enables high-throughput mining of quantitative image features from preoperative medical imaging to improve diagnostic, classification, prognostic, and predictive accuracy (25). Nevertheless, few studies regarding radiomics for accurate pre-surgical prediction of DMDG expression in LGG have been reported.

In this study, we used gene methylation, and transcriptomic and radiomics data to develop a novel LGG categorization strategy. It might be useful to optimize the individualized therapy decision and thus improve the outcomes of glioma patients.

## Methods

All methods are described in **Supplementary Methods**.

## Results

### Identification of DNA Methylation-Driven Differentially Expressed Genes in IDH^mut^/1p19q^codel^ and IDH^wt^/1p19q^non-codel^ Samples

**Figure 1:**
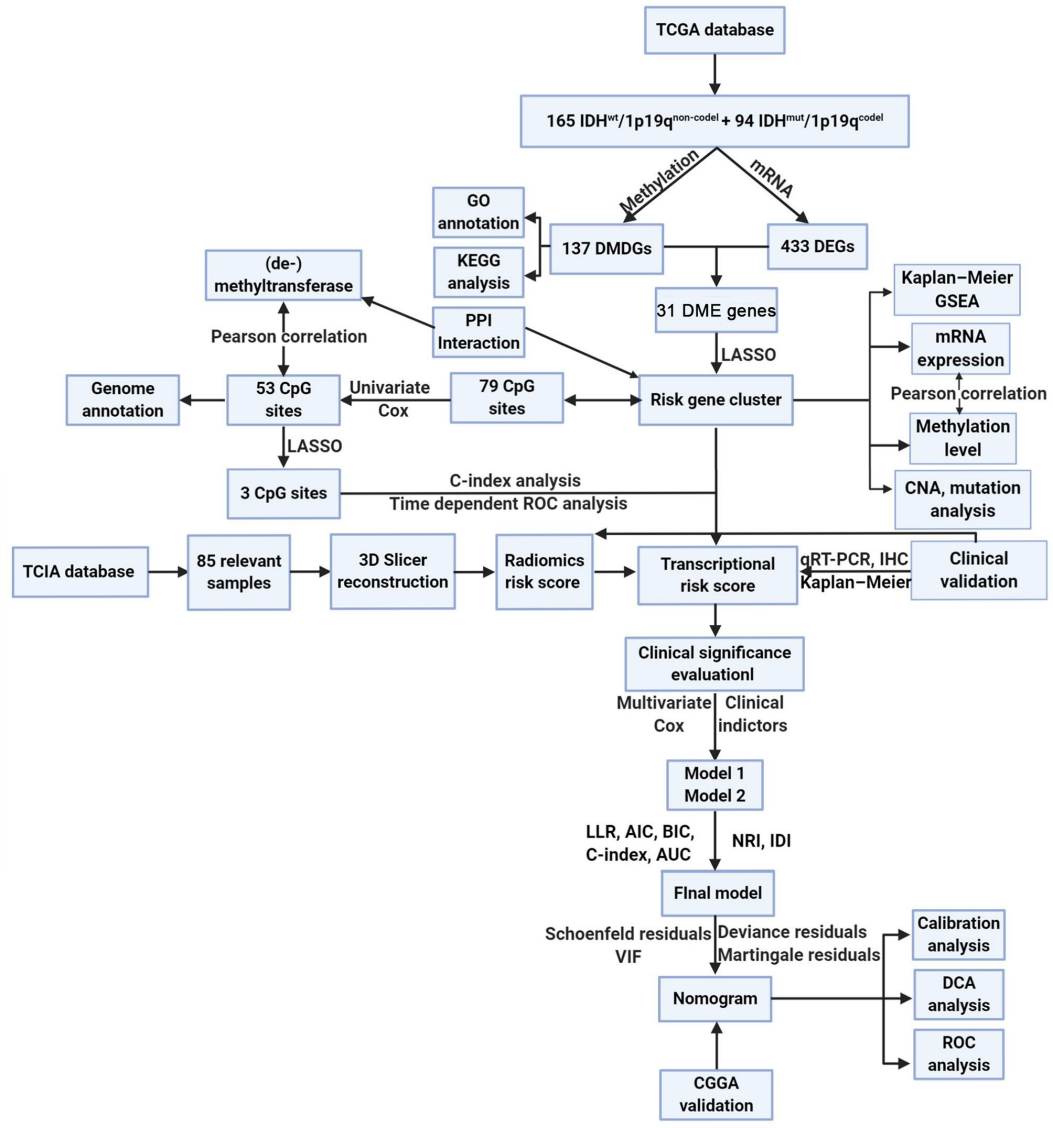
The method of this study.

**Figure 2:**
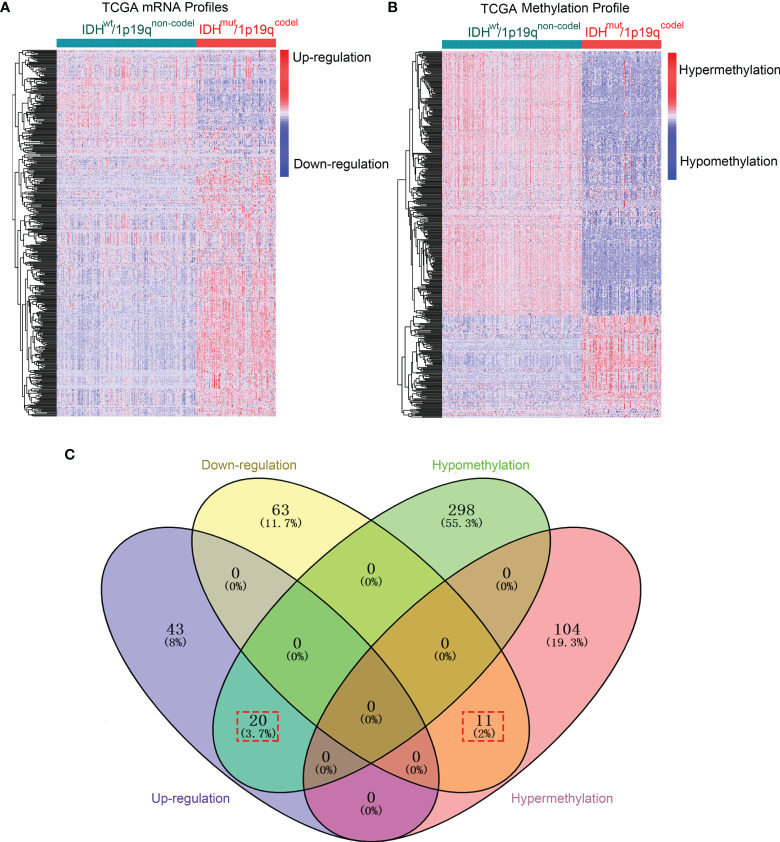
DME genes were screened in IDH/1p19q subtypes in TCGA dataset. **(A)** The hierarchical bi-clustering analysis with TCGA dataset indicated significant DEGs with 74 downregulated and 63 upregulated genes in LGGs classified by IDH and 1p/19q status. **(B)** The hierarchical bi-clustering analysis with TCGA dataset indicated significant DMDGs with 318 hypomethylated and 115 hypermethylated genes in LGGs classified by IDH and 1p/19q status. **(C)** The Venn diagram showed 31 DME genes with 20 hypomethylation that paralleled upregulation and 11 hypermethylation that paralleled downregulation. DEGs were analyzed by the *limma* package. DMDGs were analyzed by the *MethyMix* package.

The methods of this study are described in **Figure 1**. To determine differentially expressed and methylated genes, we first extracted mRNA expression and DNA methylation profiles of 259 glioma samples with WHO grade I–III from TCGA database. Patients were divided into two subgroups according to the status of IDH mutation and 1p19q integrity. The clinical and pathological characteristics between subtypes are presented in **Table S1**. Hierarchical bi-clustering was performed for IDH^mut^/1p19q^codel^ samples (*n* = 165) and IDH^wt^/1p19q^non-codel^ samples (*n* = 94). As a result, 137 candidates including 74 downregulated genes and 63 upregulated genes were selected (**Figure 2A** and **Table S2**). Subsequently, the *MethyMix* method was used to filtrate DMDGs. A total of 433 DMDGs including 318 hypomethylated genes and 115 hypermethylated genes were determined, among which the adjusted *p*-value was less than 0.05 between the hyper- and hypomethylation groups and the correlation coefficient was less than −0.3 between DNA methylation and gene expression (**Figure 2B** and **Table S3**). Afterwards, gene ontology (GO) functional annotation and Kyoto Encyclopedia of Genes and Genomes (KEGG) pathway analysis were performed to elucidate DMDG functional property (*p* < 0.05). The results demonstrated that multiple inflammation and tumor progress-related GO terms and signaling pathways were significantly enriched in IDH^wt^/1p19q^non-codel^ gliomas (**Figures S1A, B** and **Tables S4**, **S5**). The Venn diagram of DMDGs and DEGs revealed 31 DME genes, which were simultaneously hypomethylated and upregulated at the transcriptional level or hypermethylated and downregulated (**Figure 2C**).

### Establishment and Validation of the Predictive Transcriptional Risk Score

**Figure 3:**
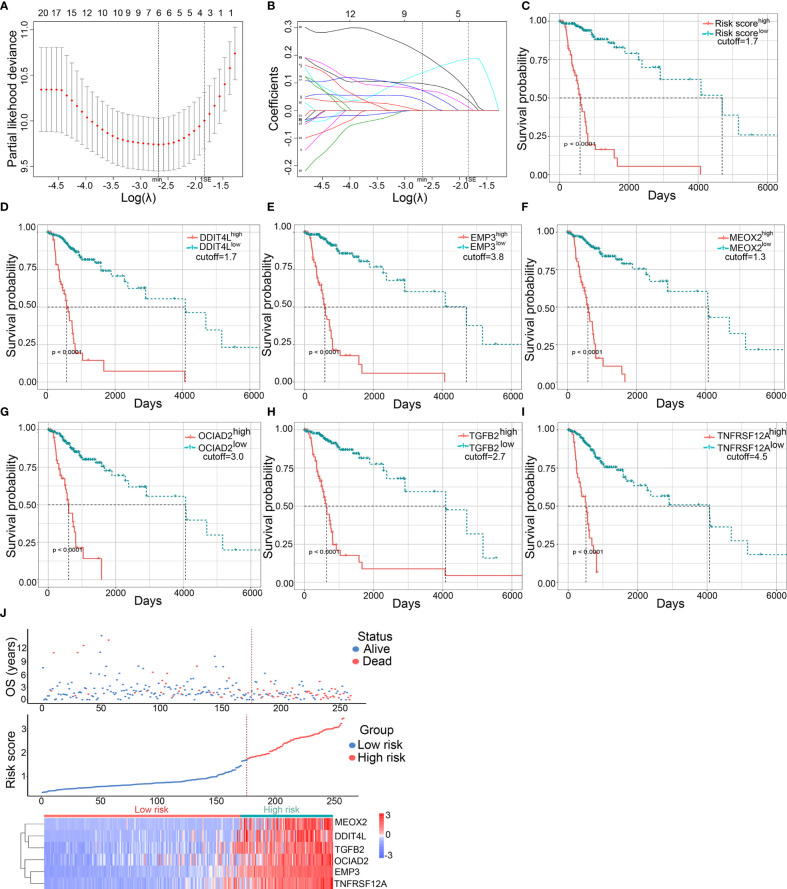
Establishment and validation of the predictive transcriptional risk score in TCGA dataset. **(A)** The risk gene cluster was selected by LASSO Cox regression with 10-fold cross-validation for tuning parameter (λ) selection in TCGA cohort, in which the vertical dashed lines showed minimum λ value and 1× standard error λ value, respectively. **(B)** The LASSO coefficient profile of all candidate genes in TCGA cohort, in which the vertical dashed lines showed minimum λ value and 1× standard error λ value, respectively. **(C–I)** The Kaplan–Meier survival curves were performed in transcriptional risk score, *DDIT4L* expression, *EMP3* expression, *MEOX2* expression, *OCIAD2* expression, *TGFB2* expression, and *TNFRSF12A* expression in TCGA dataset; the optimal cutoff value was derived from X-tile (all *p* < 0.0001, with log-rank test). **(J)** The risk factor association diagram in TCGA cohort. The results showed the blue dots in the figure representing the surviving LGG patients and the red dots representing death, and the corresponding risk gene cluster mRNA expression profiles were visualized as a heatmap. The dotted line indicated the optimal cutoff value of the mRNA risk score, in which all LGG patients were divided into two groups including 176 low transcriptional risk score samples and 83 high transcriptional risk score samples.

To further narrow down the scope of the candidate DME genes, the least absolute shrinkage and selection operator (LASSO) regression was performed to select the most suitable predictive variables. To this end, six candidate DME genes (named risk gene cluster) from a further LASSO Cox regression model were selected based on minimum lambda with 10-fold cross-validation. These genes included the following: DNA Damage Inducible Transcript 4 Like (*DDIT4L*), Epithelial Membrane Protein 3 (*EMP3*), Mesenchyme Homeobox 2 (*MEOX2*), Ovarian Cancer Immunoreactive Antigen Domain Containing 2 (*OCIAD2*), Transforming Growth Factor Beta 2 (*TGFB2*), and Tumor Necrosis Factor Receptor Superfamily Member 12A (*TNFRSF12A*) (**Figures 3A, B**). The transcriptional risk score predictive model was developed by adding the mRNA expression level and relevant coefficient of each gene in the LASSO regression as follows: transcriptional risk score = 0.0350970 × *DDIT4L* mRNA expression + 0.1368395 × *EMP3* mRNA expression + 0.0974575 × *MEOX2* mRNA expression + 0.0723336 × *OCAID2* mRNA expression + 0.0738469 × *TGFB2* mRNA expression + 0.2045352 × *TNFRSF12A* mRNA expression. Positive coefficients of all genes in the LASSO regression suggested that mRNA high expression levels were correlated with poor OS in LGG patients and the Kaplan–Meier (K–M) analysis was performed to confirm the relationship between transcriptional risk score, risk gene cluster expressions, and OS. The OS of high transcriptional risk score or mRNA high expression group was significantly shorter (**Figures 3C–I**). Of note, the X-tile method was utilized to distinguish the optimal cutoff value. Additionally, principal component analysis (PCA) was performed to assess the distinguished accuracy based on the DME genes. Compared with the thirty-one DME gene expression levels, the contributing rate of the first principal component was observably promoted to 76.2% using the risk gene cluster expression levels (**Figures S2A, B**). Despite the fact that the contributing rate of the first principal component was also ascending based on DNA methylation levels or the combination of methylation and expression using risk gene cluster, the clinical feasibility was inconvenient (**Figures S2C, D**). Therefore, the transcriptional risk score model depending on the expression of risk gene cluster was adopted for further analysis. As shown in the risk factor association diagram (**Figure 3J**), the blue dots in the figure represented the surviving LGG patients while the red dots represented death, and the corresponding risk gene cluster expression profiles were visualized as a heatmap. The dotted line indicated that the optimal cutoff value of transcriptional risk score, with which all LGG patients were divided into two groups including 176 low transcriptional risk score samples and 83 high transcriptional risk score samples. The results showed that along with the increasing of the transcriptional risk score, the number of deaths gradually increased as well as the mRNA expression levels of the risk gene cluster, demonstrating that the patients in the high transcriptional risk score group exhibited more severe survival and higher risk of death. To further elucidate signaling pathways underlying our risk score model, we perform the Gene Set Enrichment Analysis (GSEA) in the two groups. As shown in **Figure S3**, a wide range of signaling pathways related to the tumor immunological process were enriched in the high transcriptional risk score gliomas, indicating that signals from immune cells or its presence within the tumor might be crucial factors that affect progression and recurrence of glioma.

To confirm the consistency between methylation level and gene expression of the risk gene cluster in LGG patients, a difference analysis and a correlation analysis were performed. The results showed that the hypermethylation status and downregulation level of the risk gene cluster were coincidently enriched in the IDH^mut^/1p19q^codel^ group (**Figures S4**, **S5**) and significant negative correlation between methylation and mRNA expression could be observed (**Figure S6**). **Figure S7** also demonstrated the relative hypermethylation in the IDH^mut^/1p19^codel^ samples and the relative hypomethylation in IDH^wt^/1p19q^non-codel^ samples. Taken together, these findings indicated a consistent tendency between DNA methylation and the expression of risk gene cluster and implied a potential prediction model for gliomas.

To verify that the expression of the risk gene cluster was induced by corresponding DNA methylation alterations rather than by copy number alterations (CNAs) or mutations, the cBioPortal database and COSMIC database were used to investigate the CNA or mutation levels of the risk gene cluster. As shown in **Figure S8**, the risk gene cluster was not detected in either the top 20 CAN genes or mutation genes; moreover, the genetic mutation ratio of these genes was less than 3% in all glioma samples. These results indicated that the transcriptional regulation of the risk gene cluster was driven by DNA methylation alterations.

### Development and Validation of the Predictive Cytosine-Phosphate-Guanine Methylation Risk Score

To provide further insight into CpG methylation, the preprocessed CpG site methylation value of the risk gene cluster was selected. The correlation coefficients between CpG sites and risk gene cluster expression were calculated (**Table S6**). Subsequently, 53 CpG sites were initially filtrated based on univariate Cox regression analysis among 79 CpG sites (**Figure S9A**), and the relevant genomic information was presented using a heatmap (**Figure S9B**), which also presented a significant difference between the high and low transcriptional risk score groups (**Table S7**). The results indicated extensive hypomethylation located mainly in the CpG island, CpG shelf, CpG shore, and open sea of CpG sites in the high transcriptional risk score group, which might contribute to the upregulation of the corresponding risk gene cluster. Moreover, the correlation between 53 CpG sites and de-/methyltransferase derived from the Molecular Signatures Database (MSigDB) with GO_DEMETHYLATION and GO_METHYLATION was tested (**Figure S9C**). |*r*| > 0.7 and an adjusted *p*-value < 0.05 were set as cutoff criteria for further filtration of the de-/methyltransferase, of which the annotation was diagrammatized to present positive and negative de-/methyltransferase using a Sankey diagram (**Figures S9D, E**). Moreover, the protein–protein interaction (PPI) was performed by GeneMANIA (**Figure S9F**). The results indicated that de-/methyltransferase could participate in the regulation of the CpG sites of the risk gene cluster. **Figure S9G** demonstrated that 53 CpG sites were mainly distributed in promoter regions (60.4%), which included 1500 bp upstream of the transcriptional start site (TSS 1500) (20.8%), TSS 200 (13.2%), the 5’-untranslated region (5’UTR) (9.4%), and the first exon (1stExon) (17.0%). Interestingly, the 48 (90.57%) of hypomethylation CpG sites were also mainly distributed in the promoter region (63.8%) among 53 CpG sites. Therefore, we can speculate that the upregulation of risk gene cluster expression could appear due to the hypomethylation of CpG sites in the promoter regions of the corresponding genes under the effect of de-/methyltransferase.

### Assessment Transcriptional Risk Score and CpG Methylation Risk Score

To further contrast with transcriptional risk score in the accurate prediction of patient outcomes, LASSO regression was used to narrow down the candidate CpG sites. As a result, 3 CpG sites, namely, cg03208951, cg23344780, and cg23545105, were selected based on minimum lambda with 10-fold cross-validation (**Figures S10A, B**). The CpG risk score predictive model was developed by adding the product of the CpG methylation level and relevant coefficient of CpG site in the LASSO regression as follows: CpG risk score = (−1.0703883 × cg03208951) + (−1.7594301 × cg23344780) + (−0.4950028 × cg23545105). Afterwards, the 6-gene transcriptional risk score model and 3-CpG methylation risk score model were further assessed by concordance index (C-index) and time-dependent receiver operating characteristic (ROC) analysis. The results demonstrated that the prediction accuracy of about 3 years of transcriptional risk score was higher than CpG risk score and the long-term prediction accuracy was almost identical (**Figures S10C–F** and **Table S8**). Although the long-term prediction accuracy was not statistically different, the prognosis of LGG patients was different and some IDH^wt^/1p19q^non-codel^ LGG patients experienced rapid recurrence in the short term (8). Therefore, the transcriptional risk score model was selected as the most efficient prediction methods and was used to establish the nomogram.

### Evaluation of Clinical Significance of the Model in TCGA Database

**Figure 4:**
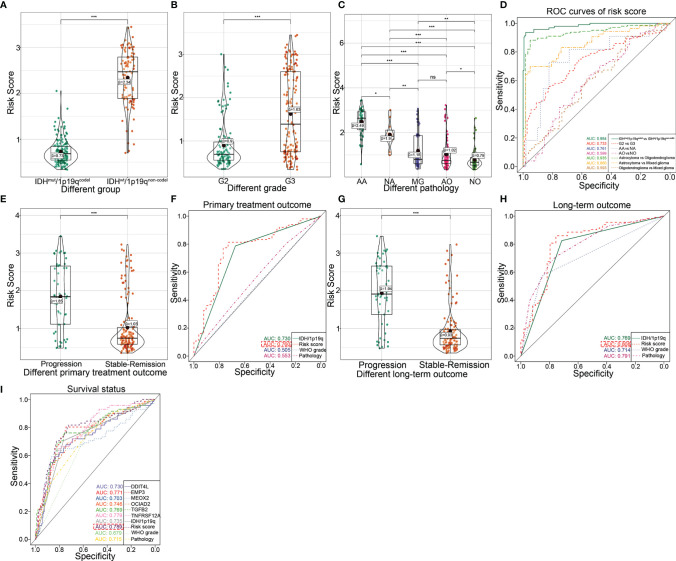
Clarifying the efficiency of the transcriptional risk score and indicating the pathological subtypes, therapy reaction, and patient survival in TCGA dataset. **(A)** The differential distribution of the transcriptional risk score in IDH^wt^/1p19q^non-codel^ and IDH^mut/^1p19q^codel^ based on TCGA cohort (***p < 0.001, with *t* test). **(B)** The differential distribution of transcriptional risk score in G2 and G3 based on TCGA cohort (***p < 0.001, with *t* test). **(C)** The differential distribution of transcriptional risk score in pathological subtypes based on TCGA cohort (***p < 0.001, **p < 0.01, *p < 0.05, and ns refer to not significance with *t* test). **(D)** The ROC curves analysis of transcriptional risk score for the IDH/1p19 group, pathological subtypes, and WHO grades based on TCGA cohort. **(E)** The differential distribution of the transcriptional risk score in different primary treatment outcomes based on TCGA cohort (***p < 0.001, with *t* test). **(F)** The ROC curve analysis of primary treatment outcome using IDH/1p19q, transcriptional risk score, WHO grades, and pathological subtypes based on TCGA cohort, respectively. **(G)** The differential distribution of the transcriptional risk score in different long-term treatment outcomes based on TCGA cohort (***p < 0.001, with *t* test). **(H)** The ROC curve analysis of long-term treatment outcome using IDH/1p19q, transcriptional risk score, WHO grades, and pathological subtypes based on TCGA cohort, respectively. **(I)** The ROC curve analysis of survival status using six DME genes, IDH/1p19q, transcriptional risk score, WHO grades, and pathological subtypes based on TCGA cohort, respectively.

We further investigate the predictive accuracies of the transcriptional risk score indicating the pathological subtypes, therapy reaction, and patient survival by using TCGA database. The results showed that the transcriptional risk score was markedly elevated in IDH^wt^/1p19q^non-codel^ gliomas (**Figure 4A**). Consistently, an increased transcriptional risk score could also be observed in WHO grade III gliomas compared to those with lower WHO grade (**Figure 4B**). Interestingly, we also found that the transcriptional risk score was significantly increased in anaplastic astrocytoma (AA), which is considered to be more malignant and undergoes transition to glioblastoma more frequently, compared to other pathological subgroups of LGG (**Figure 4C**). Moreover, the results of ROC analysis indicated that our transcriptional risk score had encouraging sensitivity and specificity for distinguishing IDH/1p19q subtypes, WHO grades, and particular pathology subtypes, especially for discrimination of astrocytoma from oligodendroglioma or mixed glioma (MG) (**Figure 4D**). However, no significant difference could be observed between MG and anaplastic oligodendroglioma (AO) (**Figures 4C, D**). Next, the predictive efficiency of our transcriptional risk score model in primary or long-term treatment gliomas was investigated. According to our data, the transcriptional risk score was significantly increased in advanced gliomas as opposed to the stable-remission ones (**Figure 4E**). Also, the ROC analysis indicated that our transcriptional risk score was significantly more efficient for treatment prediction than WHO grade or pathological classification in short-term outcomes, but the AUC had no significant statistical difference between the transcriptional risk score and IDH/1p19q (**Figure 4F**). The transcriptional risk score was also significantly increased in advanced gliomas as opposed to the stable-remission ones in long-term outcomes (**Figure 4G**). Moreover, the ROC analysis indicated that our transcriptional risk score was significantly more efficient for treatment prediction than WHO grade or IDH/1p19q in long-term outcomes, but the AUC had no significant statistical difference between transcriptional risk score and pathology (**Figure 4H**). Finally, we used ROC curve to demonstrate that the transcriptional risk score model was more efficient than the calculation based on the single indicator to patient survival (**Figure 4I**). Taken together, our transcriptional risk score model showed high sensitivity and specificity and could be used as a reliable prognostic prediction model in glioma.

### Development and Validation of the Predictive Radiomics Risk Score

**Figure 5:**
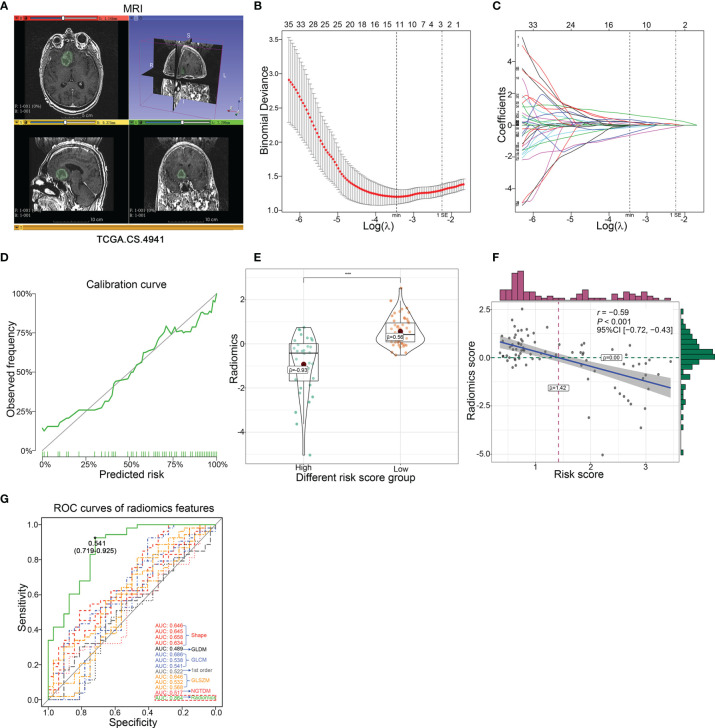
Development and validation of the predictive radiomics risk score in TCGA dataset. **(A)** The representative MRI image derived from TCGA.CS.4941 reconstructed by 3D Slicer based on TCIA. **(B)** The radiomics features were selected by LASSO logistic regression with10-fold cross-validation for tuning parameter (λ) selection in TCGA cohort, in which the vertical dashed lines showed minimum λ value and 1× standard error λ value, respectively. **(C)** The LASSO coefficient profile of all candidate radiomics features in TCGA cohort, in which the vertical dashed lines showed minimum λ value and 1× standard error λ value, respectively. **(D)** The calibration curve of radiomics risk score. **(E)** The difference of radiomics risk score in different transcriptional risk score groups with an optimal cutoff of 1.7 calculated by X-tile (***p < 0.001, with *t* test). **(F)** The correlation presented significant negative correlation between radiomics risk score and transcriptional risk score in TCGA cohort (*p* < 0.001, with Pearson correlation). **(G)** The ROC curve analysis of the transcriptional risk score group using 13 radiomics features and radiomics risk score, respectively.

It should be noted that this transcription risk score can only be evaluated after surgical resection and therefore cannot be used for pre-surgical evaluation of malignancies of LGG. Thus, in addition to the transcription risk model, a non-invasive pre-operation quantification method should be established. To this end, we used a radiomics method to explore the related features with transcription risk score and further estimate the risk score level for LGG patients in pre-surgery (**Figure 5A**). Preprocessed contrast-enhanced MR images of 85 patients with pathological diagnosis and continuous follow-up were used to identify the most correlated radiological features. All of the 107 radiological signatures with intra-class correlation coefficient >0.80 were enrolled to establish a radiomics risk score model. The predictive model was established by adding the product of the radiomics feature value and relevant coefficient of each radiomics features in the LASSO regression based on minimum lambda with 10-fold cross-validation (**Figures 5B, C**). Finally, 13 radiomics features were selected and the radiomics risk score was calculated as follows: Radiomics risk score = 0.1967277 × Voxel Volume + 0.0086076 × Mesh Volume + (−0.1419602 × Sphericity) + 0.1840789 × Maximum 2D Diameter Column + (−0.5034319 × Large Dependence High Gray Level Emphasis) + 0.6774184 × Inverse Difference Moment Normalized + (−0.0740082 × Inverse Variance) + (−0.6442577 × Cluster Prominence) + (−0.0165486 × Skewness) + 0.0521741 × Gray Level Non Uniformity_GLSZM_ + (-0.0176127 × Large Area High Gray Level Emphasis) + 0.3468764 × Zone Entropy + 0.1971442 × Strength. We then verified a suitable calibration using the calibration curve analysis. The solid straight line (the 45-degree line) showed an ideal prediction radiomics model, and the broken lines represented the observed radiomics model, in which a closer fit to the dashed line means a better prediction model, and the result showed a satisfying consequence of this model and indicated that the radiomics risk score had a more favorable fitting to the transcriptional risk score (**Figure 5D**). Importantly, the radiomics risk score showed a statistically significant negative correlation with the transcriptional risk score model (**Figures 5E, F**). Moreover, ROC analysis also indicated that the radiomics risk score model exhibited remarkably improved sensitivity and specificity compared to the usage of the single radiomics feature (**Figure 5G**). Collectively, we analyzed 85 MR post-contrast T1-weighted images of LGG patients and identified 13 transcription risk score-specific radiomic signatures, and these results demonstrated that the radiomics-dependent model could be used as a dependable method for pre-operational assessment of the transcriptional risk score.

### Clinical Validation of the Risk Gene Cluster

**Figure 6:**
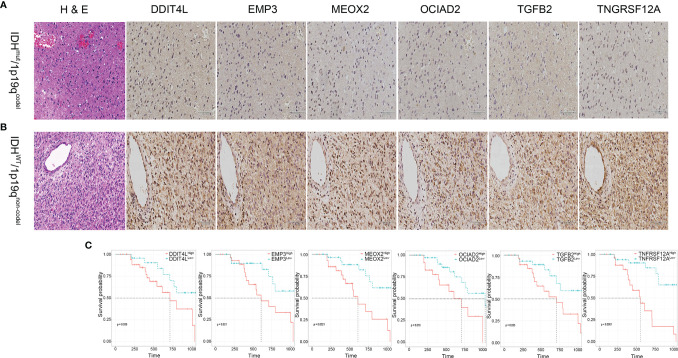
Validation of the transcriptional risk score using IHC staining in clinical samples. **(A, B)** Representative IHC images of risk gene cluster in glioma tissues samples. Upper panel, IDH^mut^/1p19q^codel^. Lower panel, IDH^wt^/1p19q^non-codel^. **(C)** The Kaplan–Meier survival analysis for risk gene cluster derived from *DDIT4L* expression, *EMP3* expression, *MEOX2* expression, *OCIAD2* expression, *TGFB2* expression, and *TNFRSF12A* expression classified by IHS using our clinical samples, respectively (all *p* < 0.05, with log-rank test).

**Figure 7:**
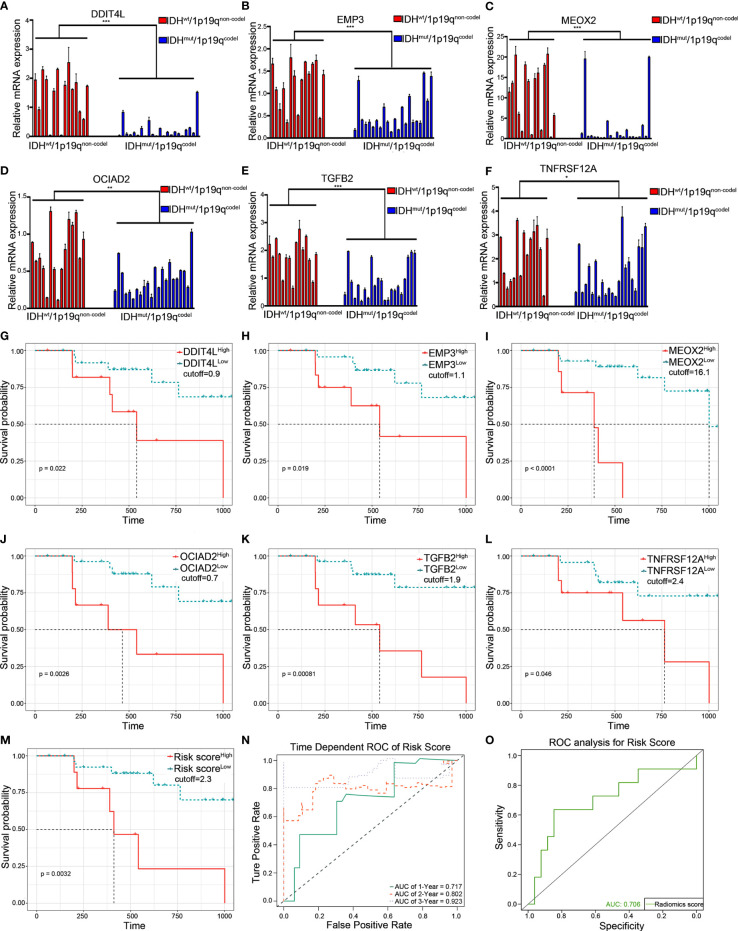
Validation of the transcriptional risk score and radiomics risk score using qRT-PCR in clinical samples. **(A–F)** qRT-PCR analysis for measuring the relative mRNA expression of risk gene cluster in 37 LGG tumor tissues grouped by IDHmut/1p19qcodel and IDHwt/1p19qnon-codel (***p < 0.001, **p < 0.01, and *p < 0.05, with *t* test, *n* = 3). **(G–M)** The Kaplan–Meier survival curves were performed in transcriptional prediction risk score, *DDIT4L* expression, *EMP3* expression, *MEOX2* expression, *OCIAD2* expression, *TGFB2* expression, and *TNFRSF12A* expression; the optimal cutoff value was derived from X-tile using qRT-PCR data in our clinical samples, respectively (all *p* < 0.05, with log-rank test). **(N)** The time-dependent ROC curve analysis for transcriptional risk score during 1, 2, and 3 years in the clinical cohort. **(O)** The ROC curve analysis of radiomics risk score in the clinical cohort. qRT-PCR, quantitative RT-PCR.

To explore the consistency of the risk gene cluster in clinical samples, immunohistochemistry (IHC) staining was performed using 61 glioma samples to address the expression level of the risk gene cluster. The results indicated that the risk gene cluster was significantly enriched in the IDH^wt^/1p19q^non-codel^ group compared with their corresponding counterparts (**Figures 6A, B**). Moreover, the K–M analysis indicated that the higher immunohistochemical score (IHS) of the risk gene cluster correlated with poor prognosis for glioma patients, which is consistent with the results obtained from TCGA database (**Figure 6C**). The quantitative reverse transcription PCR (qRT-PCR) analysis was also performed to quantify the expression levels of the risk gene cluster by using 37 glioma samples with complete radiographic data and survival data (22 IDH^mut^/1p19q^codel^ samples and 15 IDH^wt^/1p19q^non-codel^ samples) and a non-tumor tissue derived from epilepsy patient used as a control. Consistently, the results showed that the risk gene cluster was significantly elevated in the IDH^wt^/1p19q^non-codel^ group (**Figures 7A–F**). Similar to the results from IHC, K–M analysis also demonstrated prolonged OS for the patients with lower expression of these risk genes (**Figures 7G–M**). To further validate the reliability of our model, the transcription risk score model and radiomics model were calculated using clinical samples. The transcriptional risk score exhibited satisfying AUCs of 1-, 2- and 3-year OS (0.717, 0.802, and 0.923, **Figure 7N**) based on qRT-PCR analysis, and the radiomics score also presented appropriate AUC (0.706) based on pre-surgical MRIs (**Figure 7O**). Of note, the cutoff values were recalculated by X-title. Taken together, these results indicated that our transcriptome model and radiomics model showed reasonably good reliability in our clinical cohort.

### Establishment and Assessment of the Comprehensive Nomogram in TCGA Dataset

Based on our previous results, the transcriptional risk score was the most appropriate and accurate method compared to the others. Therefore, we adopted transcriptional risk score combined with clinical indicators to establish a novel nomogram for pre-surgical assessment of patient survival and therapy reaction. To this end, univariate Cox regression followed by multivariate Cox regression were performed to identify the most significant independent risk/protective factors. As a result, transcriptional risk score presented the most significant hazard ratio (HR) (HR = 2.94, 95% CI: 1.60–5.42, *p* < 0.01); in addition, patient age was also confirmed to be an independent risk factor with an HR of 2.55 (95% CI: 1.76–3.71, *p* < 0.001) (**Table 1**). Therefore, patient age was enrolled in addition to the transcriptional risk score to establish the prediction nomogram.

**Table 1:** Univariate and multivariate Cox regression of variables.

	Univariate Cox Regression			Multivariate Cox Regression		
Variables	HR	95% CI	*p*	HR	95% CI	*p*
Age, per 1 SD, years	2.93	2.19–3.92	**<0.001**	2.55	1.76–3.71	**<0.001**
Risk score, per 1 SD	3.30	2.56–4.24	**<0.001**	2.94	1.60–5.42	**<0.001**
Gender						
Female	1 (Ref)					
Male	1.23	0.77–1.98	0.388			
Group						
IDH^mut^/1p19q^codel^	1 (Ref)			1 (Ref)		
IDH^wt^/1p19q^non-codel^	7.66	4.54–12.92	**<0.001**	0.74	0.19–2.80	0.655
WHO Grade						
G2	1 (Ref)			1 (Ref)		
G3	4.91	2.54–9.48	**<0.001**	2.19	0.56–8.61	0.264
Pathology						
AA	1 (Ref)			1 (Ref)		
NA	0.59	0.18–1.96	0.389	2.64	0.42–16.71	0.302
MG	0.34	0.17–0.67	**0.002**	1.62	0.73–3.60	0.233
AO	0.33	0.18–0.59	**<0.001**	1.00	0.47–2.15	0.996
NO	0.08	0.03–0.18	**<0.001**	0.81	0.17–3.89	0.796

SD, standard deviation; IDH, isocitrate dehydrogenase; 1p19q, the chromosome 1p and 19q; G2 and G3, WHO grade 2 and grade 3; AA, anaplastic astrocytoma; NA, not otherwise specified astrocytoma; MG, mixed glioma; AO, anaplastic oligodendroglioma; NO, not otherwise specified oligodendroglioma; HR, hazard ratio. Bold means the significant statistical difference.

Given that the age is well known to affect the methylation status of genes (26), the interaction between age and transcriptional risk score was verified *via* the interaction test. The result showed that the interaction was statistically significant, indicating that patient age might affect the expression of the risk gene cluster (**Table 2**, *p* for interaction = 0.033). Therefore, we performed further stratified analyses to eliminate this ambiguous association (**Table 2**). Two hundred and fifty-nine patients were divided into 4 subgroups according to quartile categories of age, and the transcriptional risk score was divided into 3 subgroups according to tertile categories of risk score: Q1 (17–37 years old), Q2 (38–48 years old), Q3 (49–58 years old), and Q4 (59–87 years old), and low risk (Q1, transcriptional risk score: 0.3376742–0.6905997), median risk (Q2, transcriptional risk score: 0.6943530–1.6467377), and high risk (Q3, transcriptional risk score: 1.6655889–3.4480996); the median of each subgroup was used for statistical comparison (**Table 2**). Significant differences were observed in all age subgroups (total HR: 2.371, 7.279, 5.285, and 2.078, *p* for trend: 0.038, <0.001, <0.001, and 0.002). The results demonstrated that the mortality risk of LGG patients was gradually elevated along with the increase of transcriptional risk score in each age subgroup. In particular, we found that the HR of transcriptional risk score showed an inverted U-shaped distribution along with the increase of age with the peak value appearing in the Q2 subgroup (**Figure S11A**). Collectively, these data suggested that enrichment of the risk gene cluster implied the highest risk of death of LGG in 35–45 years.

**Table 2:** Stratification analysis of age and risk score.

Variables		Risk score (Median) adjusted HR (95%)			Total HR (95% CI)	*p* for trend	*p* for interaction
	*N*	Q1 (0.55284)	Q2 (0.86109)	Q3 (2.54137)			
Age, years (Median)							**0.033**
Q1 (31.5)	68	1 (Ref)	0.840 (0.074–9.526)	4.855 (0.866–27.210)	2.371 (1.048–5.360)	**0.038**	
Q2 (43.0)	64	1 (Ref)	0.00 (0.000–5.828*10^165^)	25.936 (3.240–207.641)	7.279 (2.659–19.931)	**<0.001**	
Q3 (54.0)	62	1 (Ref)	3.196 (0.329–31.028)	41.648 (4.680–370.796)	5.285 (2.549–10.961)	**<0.001**	
Q4 (64.0)	65	1 (Ref)	1.220 (0.313–4.761)	4.212 (1.232–14.401)	2.078 (1.322–3.265)	**0.002**	

HR, hazard ratio. Bold means the significant statistical difference.

Next, we used variance inflation factor (VIF) to test the collinearity, which leads to some weaknesses such as unstable parameter estimation, unreliable models, and weak predictive ability. Given the result of the interaction test between age and transcriptional risk score, we defined the interaction term: age × risk score (A.R.). The VIFs of age, risk score, and A.R. were 3.918, 20.954, and 28.425, respectively, which indicated that the collinearity could exist between risk score and A.R. However, after mean-centering (each independent variable minus the corresponding average), the VIFs of age, risk score, and A.R. were 1.119, 1.231, and 1.106, respectively, pointing out the nonessential collinearity (27). Afterwards, two models were respectively constructed to assess whether adding A.R. can increase the performance of the model. As shown in **Table 3**, there were no significant increase in terms of log-likelihood ratio (LLR), C-index, Akaike information criterion (AIC), Bayesian information criterion (BIC), and AUC of time-dependent ROC. Compared with model 2, the net reclassification improvement (NRI) and integrated discrimination improvement (IDI) of model 1 also did not improve (**Table 3**, all *p* > 0.05). Therefore, the age and risk score were included in the nomogram based on simplicity and efficiency.

**Table 3:** The filtration of models.

Variables	Model 1		Model 2	
		*P*		*p*
HR of age, per 1 SD, years	2.535 (1.806–3.557)	**<0.001**	5.166 (2.436–10.957)	**<0.001**
HR of risk score, per 1 SD	2.858 (2.201–3.713)	**<0.001**	11.854 (3.082–45.594)	**<0.001**
HR of A.R., per 1 SD	——		0.208 (0.049–0.878)	**0.033**
LLR	125.7	**<0.001**	130.3	**<0.001**
C-index				
Corrected	0.870	——	0.869	——
Uncorrected	0.873	——	0.873	——
AIC	531.012	——	528.406	——
BIC	535.537	——	535.194	——
AUC				
1 year	0.888	——	0.891	——
2 years	0.922	——	0.931	——
5 years	0.966	——	0.976	——
NRI (model 2 vs model 1)				
1 year	0.030 (−0.173–0.318)			0.585
3 years	0.394 (−0.126–0.537)			0.08
5 years	0.614 (−0.385–1.578)			0.113
IDI (model 2 vs model 1)				
1 year	−0.021 (−0.047–0.004)			0.126
3 years	0.035 (−0.002–0.082)			0.06
5 years	0.061 (−0.020–0.191)			0.100

SD, standard deviation; LLR, log-likelihood ratio; AIC, Akaike information criterion; BIC, Bayesian information criterion; NRI, the net reclassification improvement; IDH, integrated discrimination improvement; C-index, Concordance index; AUC, area under curve; A.R., age × risk score; HR, hazard ratio. Bold means the significant statistical difference.

**Figure 8:**
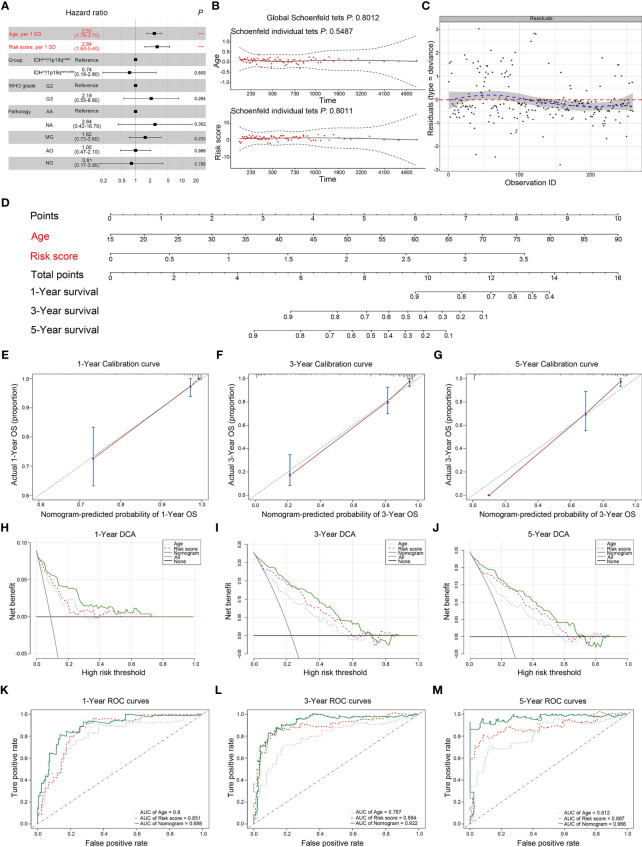
Establishment and assessment of the comprehensive nomogram in TCGA dataset. **(A)** Univariate and multivariate analyses of the transcriptional risk score, clinical factors, and pathological subtypes with OS. The statistical significance level was indicated by different colors; red indicated statistical significance, and black indicated no significance. **(B)** The Schoenfeld residual suggested that this model met the PH. The Schoenfeld model residuals of age and transcriptional risk score were plotted to obtain a preliminary assessment in which these predictive indicators should be enrolled in the nomogram. **(C)** The Deviance residuals test indicated that there were no outliers. **(D)** Comprehensive nomogram including age and transcriptional risk score was established to predict 1-, 3-, and 5-year OS probability in LGGs. **(E–G)** The calibration curves of 1, 3, and 5 years showed more appropriate calibration ability in TCGA cohort, in which the blue dotted lines represented the ideal predictive model, and the red solid line represented the nomogram model. **(H–J)** The DCA curves showed a comparable net benefit if the threshold probability for a patient or a doctor was within a range from 0 to 0.80 during 1, 3, and 5 years. The *y*-axis represented the net benefit. The *x*-axis represented the predicted OS probability. The oblique smooth solid line represented a type of hypothesis in which all patients survive at a corresponding time. The horizontal smooth solid line represented a type of hypothesis in which none of the patients survive for more than 1 year. **(K–M)** The time-dependent ROC curve analysis for the nomogram and single indicator during 1, 3, and 5 years in TCGA cohort, respectively. ***P < 0.001.

The forest plot presented the age and risk score as independent risk factors (**Figure 8A**). The Schoenfeld residual test showed that all of the variables met equally proportional hazards (PH) assumption (**Figure 8B**) and there were no outliers based on the Deviance residual test (**Figure 8C**). The Martingale residuals demonstrated the linear relationship between age and transcriptional risk score with the logit transformation value of the hazard and the restricted cubic spline (RCS) analysis also verified (**Figures S11B–E**). Considering all the previously mentioned significant predictive factors, we established a comprehensive nomogram including age and transcriptional risk score (**Figure 8D**). We have also calculated the uncorrected and corrected C-index, which were 0.873 and 0.870, respectively (**Table 3**). The calibration curves of 1 year, 3 years, and 5 years indicated a suitable calibration efficiency while a closer fitness to the dashed line indicates a better prediction performance (**Figures 8E–G**). The decision curve analysis (DCA) was used to assess the clinical applicability of nomogram and a net benefit for diverse prediction models at different threshold probabilities by adding the benefits and minimizing the harms. As demonstrated by the favorable probability, the comprehensive nomogram showed better net benefit than age and risk score (**Figures 8H–J**). Moreover, the time-dependent ROC curves verified that the prediction performance of the nomogram was gradually elevated along with the increase in time and also was better compared to the single index (**Figures 8K–M**). To create an intuitive application, the nomogram was further developed into a web version and could be dynamically operated online: https://drw576223193.shinyapps.io/Nomo/. Thus, the comprehensive nomogram was established according to the multiple prognostic factors that surpassed each single factor taken alone. The nomogram could help clinicians make more accurate assessment of patient prognosis.

### Validation of Nomogram Using the CGGA Dataset

To confirm the reliability of the comprehensive nomogram, the gene expression profiles were extracted from the CGGA dataset and then used for further model validation. Consistently, the expression level of the risk gene cluster was increased in IDH^wt^/1p19q^non-codel^ samples and correlated with more severe prognosis of glioma patients (**Figures S12**, **S13**). Furthermore, the calibration curves of the comprehensive nomogram for the possibility of 1-, 3-, and 5-year OS displayed obvious concordance between the predicted results and observations (**Figures S14A–C**). In addition, the uncorrected and corrected C-index were 0.837 and 0.831, respectively, indicating that the comprehensive nomogram had an appropriate discrimination in the CGGA cohort. Similar to the results from the TCGA cohort, the ROC analysis demonstrated that our nomogram exhibited gratifying sensitivity and specificity on prognostic prediction with the AUCs of 0.845, 0.900, and 0.883 for the 1-, 3-, and 5-year survival, respectively (**Figure S14D**). Collectively, the results demonstrated that the comprehensive nomogram model validated both the training and the validation cohorts well.

## Discussion

Although a wide range of molecular biomarkers, most notably IDH mutations and 1p/19q integrity, have allowed for a more granular method with which to categorize glial tumors with clear prognostic implications, it is also inadequate for stratification of risk for gliomas simply according to IDH and 1p19q status (9, 28). Aberrant alteration of DMDGs in the promoter regions can be detected and have been proven to be associated with oncogenic transformation and prognosis of patients (18, 29). The DMDGs that could be used for survival prediction and clinical management of LGG patients remain unknown. Therefore, development of reliable biomarkers based on DMDGs in LGGs becomes an urgent need.

In the present study, 259 patients from TCGA dataset with pathological LGG diagnosis were stratified into two groups according to IDH mutation and 1p19q chromosomal integrity. With statistical screening, six DME genes, namely, *DDIT4L*, *EMP3*, *MEOX2*, *OCIAD2*, *TGFB2*, and *TNFRSF12A*, were identified as the risk gene cluster that revealed similar survival patterns and its’ downregulation remarkably correlated with prolonged survival in the IDH^mut^/1p19q^codel^ LGG subtype. Additionally, investigation of the potential CpG sites of risk gene cluster demonstrated 32 CpG sites distributed in promoter regions (60.4%) and 30 CpG sites among these were hypomethylation (93.75%), which was consistent with the previous studies (14, 30). Moreover, the PPI network showed a complex interaction between risk gene cluster and the de-/methyltransferase. These results indicated that the regulation of the risk gene cluster was related to these de-/methyltransferase *via* reduction of the corresponding CpG site methylation probably through co-expression and physical interaction. Importantly, risk gene cluster showed better performance compared to CpG risk score, indicating that mRNA expression profiles could be more suitable for prediction of patient survival in LGGs. Notably, previously reported predictable models showed nonnegligible limitation because of the ignorance of the interaction effects of age and methylation levels (29). Therefore, we found that the statistical difference of the interaction of age and risk score was statistically significant (*p* for interaction = 0.033), indicating that patient age could affect the level of methylation. According to stratification analysis, we found firstly that the HR of risk score presented an inverted U-shaped distribution along with the increase of age, in which the peak value was detected at the 38- to 48-year-old subgroup. Thus, we speculated that patients with LGG could present the highest risk of death due to the upregulation of the risk gene cluster; the risk gene cluster was more suitable to assess the prognosis in the 38- to 48-year-old subgroup. Taken together, the established novel multi-omics models are helpful in clinical management of LGGs, particularly in those with ambiguous pathological signatures.

Among these six DME genes, *DDIT4L* and its homolog *DDIT4* are upstream inhibitors of mammalian target of rapamycin (mTOR) in partial tissues and cell models; mTOR responds to various stimuli such as growth factors, cellular energy status, oxygen concentrations, and stress to control cell metabolism and growth (31, 32). Koga et al. (33). demonstrate that the promoter methylation level of *DDIT4L* is predominantly detected in advanced-stage tumors and it can be useful for evaluating melanoma tumor progression. Ozdemir et al. (34). find tumor suppressor genes, including *DDIT4L*, that are significantly elevated in the metformin and pioglitazone combination-treated anaplastic thyroid cancer cells. However, the expression and methylation level of *DDIT4L* in glioma are barely reported. *EMP3* is a member of the peripheral myelin protein 22-kDa (*PMP22*) gene family, and it is demonstrated that reintroduction in *EMP3*-deficient cancer cells inhibits colony formation and tumor growth in xenografts (35). Hong et al. (36). find that SK-BR-3 cells exhibit remarkable proliferation and invasion inhibitory effects *in vitro* when *EMP3* is knocked down by shRNA, which demonstrates that *EMP3* could function as an oncogene in human breast cancer. However, transcriptional silencing of *EMP3* in neuroblastoma and glioma cell lines is associated with aberrant methylation at exon 1 of *EMP3*; hypermethylation level is associated with poor 2-year survival and neuroblastoma-caused mortality, indicating a tumor-suppressing function (37). These contradicting results require further experimental validation. *MEOX2* belongs to the homeobox gene family and has been established as a growth arrest-specific homeobox by cyclin-dependent kinase inhibitor p21 and p16 activation (38). The dual role of *MEOX2* is also reported in recently published study. Bao et al. (39) find a cluster risk gene signature including *MEOX2*, which is related to shorter prognosis in a cohort of mesenchymal glioblastomas. Conversely, *MEOX2* has been reported to downregulate in glioblastoma cell lines compared to normal astrocytes; thus, it could be an antioncogene (40). *OCIAD2* is an immunoreactive protein with an unclear function, the expression of which is diverse in different cancers (41). The expression of *OCIAD2* was highly expressed in the invasive adenocarcinoma than in the *in situ* adenocarcinoma in lung cancer, whereas the expression level is significantly reduced in liver cancer and gastric stroma carcinoma, when compared with that in the corresponding normal tissues (42, 43). In glioma, the role and function of *OCIAD2* also remain controversial. Downregulation of *OCIAD2* is detected in glioblastoma rather than in anaplastic astrocytoma, and hypermethylation of *OCIAD2* in glioblastoma is related to a dramatic reduction in the expression level of *OCIAD2* (44, 45). On the other hand, Nikas et al. (46). have reported that *OCIAD2* is overexpressed in gliomas that have a poor prognosis. *TGFB2*, a member of the transforming growth factor-β family, is specifically overexpressed in highly aggressive glioma and is involved in brain tumor development (47). Enriched *TGFB2* expression levels are usually observed in the later stages of tumor progression and in up to 95% of high-grade gliomas, which initiates an autocrine loop to promote its own expression and enable oncogenic activity (48). Besides, this cytokine also has a dual role in oncogenesis, which can act as either a tumor suppressor or as a tumor promoter in various conditions and tumor stage (49, 50). *TNFRSF12A* is the smallest member of the TNF superfamily of receptors; it contains a short cytoplasmic demise domain and has been reported to be elevated in different cancers (51–53). It is reported that *TNFRSF12A*/*TNFRSF12* (only known ligand for *TNFRSF12A*) signaling is related to tumor metastasis and progression, as well as immune surveillance and angiogenesis (54). Sequencing analysis has confirmed that *TNFRSF12A* mRNA levels are low in normal brain and increase with glioma grade (55). Moreover, *TNFRSF12A* is a strong prognostic predictor for patients diagnosed with oligodendroglial or astrocytic tumors (56). Interestingly, it is reported that only IDH1/2 wild-type gliomas (59% GBMs and 41% LGGs) highly expressed *MEOX2* compared with IDH1/2-mutated gliomas in TCGA dataset. *EMP3* is overexpressed in oligodendroglia tumors with integrity of 1p and 19q chromosome arms (57, 58). Taken together, the dual functions of these risk genes in oncogenesis could exhibit tissue-specific expression, and transformation from tumor suppressor to tumor promoter could be presented due to epigenetic reversal in IDH-mutated/wild-type LGGs; IDH mutation results in dramatically elevated levels of 2-hydroxyglutarate (a potential oncometabolite) (59) and could influence the functions of these risk genes. Therefore, we considered that the upregulation of the risk gene cluster could be a stimulator that contributes to malignant transition in LGGs.

In this study, we developed for the first time a radiomics model using MR post-contrast T1-weighted images to assist the assessment of the level of risk gene cluster in LGGs before surgery. Eventually, 85 patients and 13 important radiomic features, namely, 4 shapes, 1 gray-level dependence matrix (GLDM), 3 gray-level co-occurrence matrices (GLCMs), 1 first order, 3 gray-level size zone matrices (GLSZMs), and 1 neighboring gray tone difference matrix (NGTDM), were included. According to our radiomics score, tumor shape features played an important role in predicting transcriptional risk score, among which the AUCs of shape features surpassed almost other features. The result is consistent with a previous study that used a random forest model to predict the presence of H3 K27M mutation in spinal cord diffuse midline gliomas and found that the maximum length of the tumor was the most important radiological feature in the model (60). In this study, we found that the radiomics risk score showed a negative correlation with the transcriptional risk score. We speculated that the negative correlation between radiomics risk score and transcriptional risk score in LGG could be affected by expression of genes, which changes the morphological feature or regional cerebral blood flow that is reflected in MRI. Tumor shape features are independent of the gray-level intensity distribution in the region of interest (ROI). The study reveals that patients with spherical tumors survive significantly longer than those with irregular tumor surface in glioblastomas, which indicate that tumors with irregular surface could be more malignant than spherical tumors (61). Texture features, including GLDM, GLCM, GLSZM, and NGTDM, are another group of widely used radiomics features based on gray-level intensity. There is a biological rationale that IDH-mutated glioma shows lower cerebral blood volume due to lower levels of hypoxia-inducible-factor 1-alpha *via* the 2-hydroxyglutarate-mediated activation of EGLN prolyl 4-hydroxylases, and present a decrease in proangiogenic signaling that is reflected as lower cerebral blood volume in perfusion-weighted MRI in comparison with IDH wild-type glioma (62). Collectively, the radiomics score also showed favorable sensitivity (92.5%) and specificity (71.9%), which will be helpful to clinicians to estimate the benefits and making individualized clinical management before the surgical resection.

Herein, we established and validated a predictable multi-omics model based on transcriptional signatures and clinical characteristics to predict patient outcomes and guide clinical decisions. Although our novel biomarker presented several advantages compared to the current diagnosis strategies, ambiguity and limitation still exist and need to be further studied. As previously described, our nomogram model derived from RNA sequencing data showed a higher precision and more favorable discrimination; however, the current methods for determination of transcriptome signatures are mainly through RNA sequencing or qRT-PCR, which are technologically complex and could only be achieved post-surgically and thus cannot be used as a pre-operational management strategy. Considering the urgent need for rapid noninvasive diagnosis methods, the radiomics risk score model was further developed. This model also showed satisfying sensitivity; however, the enrolled radiomics features cannot be transferred into visualized findings on CT/MRI. Also, the radiomics features used in this study were extracted from contrast-enhanced T1W1 MRI images. As is well known, T2-FLAIR images provide more detailed information and clear identification of infiltrating tumor edge, which is essential for maximum surgical resection (63). Therefore, more radiomics characteristics should be enrolled in subsequent studies by involving T2-FLAIR and other scanning sequences to establish more efficient radiomics models. Finally, due to the small scale of our cohort, the described multi-omics models were validated by retrospective methods, which is not sufficient to achieve a universally applicable conclusion. Large-scale multicentric prospective studies should be further performed using artificial intelligence techniques such as deep learning to improve the current models.

## Conclusions

Our novel multi-omics nomograms represented satisfying performance of LGG patients and assisted clinicians to draw up individualized clinical management.

## Data Availability Statement

The raw data supporting the conclusions of this article will be provided upon reasonable request.

## Ethics Statement

The studies involving human participants were reviewed and approved by the Scientific Ethics Committee of The First Affiliated Hospital of Xi’an Jiaotong University, Xi’an, China (approval no. 2016–18). Written informed consent for participation was not required for this study in accordance with the national legislation and the institutional requirements.

## Author Contributions

Conceptualization, WW, YW, JW, and MW. Methodology, YW and JX. Software, WW and YW. Validation, JX, XL, and AW. Formal analysis, AW. Investigation, XY, GY, and CW. Resources, XB and NW. Data curation, CD. Writing—original draft preparation, WW. Writing—review and editing, JW. Visualization, WW. Supervision, WX. Project administration, MW. Funding acquisition, JW and CD. All authors contributed to the article and approved the submitted version.

## Funding

This study was supported by the National Natural Science Foundation of China (81802502), the Project Supported by Natural Science Basic Research Plan in Shaanxi Province of China (2019JQ-958), the Fundamental Research Funds for the Central Universities (1191329177), and the Natural Science Basic Research Plan in Shaanxi Province of China (xzy012019096).

## Conflict of Interest

The authors declare that the research was conducted in the absence of any commercial or financial relationships that could be construed as a potential conflict of interest.

## Publisher’s Note

All claims expressed in this article are solely those of the authors and do not necessarily represent those of their affiliated organizations, or those of the publisher, the editors and the reviewers. Any product that may be evaluated in this article, or claim that may be made by its manufacturer, is not guaranteed or endorsed by the publisher.

## Supplementary Material

Supplementary Figure 1 The GO annotation and KEGG signaling pathway analysis in TCGA dataset. **(A)**, the GO annotation exhibited several significant terms in IDH^wt^/1p19q^non-codel^ gliomas. **(B)**, the KEGG signaling pathway demonstrated that multiple inflammation and tumor progress-related signaling pathways were significantly enriched in IDH^wt^/1p19q^non-codel^ gliomas. The GO annotation was performed by DAVID. The KEGG signaling pathway analysis was performed by ConsensusPathDB.

Supplementary Figure 2 The PCA was performed to assess the distinguished accuracy in TCGA dataset. **(A)**, the PCA presented subtype using thirty-one DME genes expression levels. **(B)**, the PCA presented subtype using risk gene cluster expression levels. **(C)**, the PCA presented subtype using risk gene cluster methylation levels. **(D)**, the PCA presented subtype using the combination of risk gene cluster expression and methylation.

Supplementary Figure 3 The GSEA in low- and high risk score group in TCGA dataset.

Supplementary Figure 4 Assessment the differential distribution of gene methylation level of the risk gene cluster in LGG patients using TCGA dataset. **(A-F)**, the differential distribution of risk gene cluster methylation level in IDH^wt^/1p19q^non-codel^ and IDH^mut^/1p19q^codel^ in TCGA cohort (all ****P*<0.001, with *t* test).

Supplementary Figure 5 Assessment the differential distribution of mRNA expression level of the risk gene cluster in LGG patients using TCGA dataset. **(A-F)**, the differential distribution of risk gene cluster mRNA expression level in IDH^wt^/1p19q^non-codel^ and IDH^mut^/1p19q^codel^ in TCGA cohort (all ****P*<0.001, with *t* test).

Supplementary Figure 6 Assessment the correlation between mRNA expression level and methylation level of the risk gene cluster in LGG patients using TCGA dataset. **(A-F)**, the correlation presented significantly negative correlation between expression and methylation of risk gene cluster in TCGA cohort (all *P*<0.001, all *r*<-0.6, with Pearson correlation).

Supplementary Figure 7 Assessment the distribution of methylation of the risk gene cluster in LGG patients using TCGA dataset. **(A-F)**, the distribution of methylation level between two mixture components in TCGA cohort, which the horizontal black bar demonstrated the relative hypermethylation in the IDH^mut^/1p19^codel^ samples and the histogram represented the relative hypomethylation in IDH^wt^/1p19q^non-codel^ samples.

Supplementary Figure 8 Verification CNA and mutation characteristics of DME genes. **(A, b)**, showed the top 20 genes with mutation and CNA in all glioma types based on cBioPortal database, respectively. **(C)**, the genetic alterations waterfall plot of the risk gene cluster which were less than 3% using cBioPortal database. **(D)**, showed the top 20 genes with mutation in all glioma types based on COSMIC database. **(E, F)**, the genetic alterations of the risk gene cluster which were less than 3% using COSMIC database.

Supplementary Figure 9 Filtration of the CpG sites of risk gene cluster in TCGA dataset. **(A)**, the volcano plot showed the result of univariate Cox regression analysis among 79 CpG sites in TCGA cohort, which the red dots represented significant 53 CpG sites with *P*<0.01. **(B)**, the heatmap showed the distribution of 53 CpG sites methylation and relevant location in genome between high- and low risk score based on TCGA cohort. **(C)**, the correlation heatmap between 53 CpG sites and de-/methyltransferase. **(D, E)**, the Sankey diagram presented the interaction between significant de-/methyltransferase and corresponding CpG sites (|*r*|>0.7 and *P*<0.05, with Pearson correlation). **(F)**, the PPI showed the interaction between significant de-/methyltransferase and risk gene cluster. **(G)**, the distribution of 53 CpG site in genome. Especially, the CpG sites were derived from Illumina Human Methylation 450 platform and preprocessed by *ChAMP* package. The PPI was analyzed by GeneMANIA.

Supplementary Figure 10 Development and validation of the predictive CpG risk score in TCGA dataset. **(A)**, the CpG sites were selected by LASSO Cox regression with10-fold cross-validation for tuning parameter (λ) selection in TCGA cohort, which the vertical dashed lines showed minimum λ value and 1 times standard error λ value, respectively. **(B)**, the LASSO coefficient profile of all candidate CpG sites in TCGA cohort, which the vertical dashed lines showed minimum λ value and 1 times standard error λ value, respectively. **(C)**, the assessment between 6-gene transcriptional risk score and 3-CpG risk score by C-index based on pec package. **(D-F)**, the time dependent ROC curves analysis showed performance of 6-gene transcriptional risk score and 3-CpG risk score during 1-, 3- and 5-years, respectively. LASSO, least absolute shrinkage and selection operator.

Supplementary Figure 11 Statistical test the suitability of age and transcriptional risk score. **(A)**, the HR of transcriptional risk score showed an inverted U-shaped distribution along with the increase of age. **(B, C)**, the Martingale residuals demonstrated the linear relationship between age and transcriptional risk score with the logit transformation value of the hazard. **(D, E)**, the RCS analysis verified the linear relationship between age and transcriptional risk score with the logit transformation value of the hazard.

Supplementary Figure 12 Assessment the differential distribution of mRNA expression level of the risk gene cluster in LGG patients using CGGA dataset. **(A-F)**, the differential distribution of risk gene cluster mRNA expression level in IDH^wt^/1p19q^non-codel^ and IDH^mut^/1p19q^codel^ in CGGA cohort (all ****P*<0.001, with *t* test).

Supplementary Figure 13 The survival plot of the risk gene cluster in LGG patients in CGGA dataset. **(A-G)**, the Kaplan-Meier survival analysis of *DDIT4L* expression, *EMP3* expression, *MEOX2* expression, *OCIAD2* expression, *TGFB2* expression, *TNFRSF12A* expression and transcriptional risk score which classified by optimal cutoff value derived from X-tile based on CGGA database, respectively (all *P*<0.001, with log-rank test). **(H)**, the risk factor association diagram in CGGA cohort. The results showed the blue dots in the figure represented the surviving LGG patients while the red dots represented death, and the corresponding risk gene cluster mRNA expression profiles were visualized as a heatmap. The dotted line indicated that the optimal cut-off value of mRNA risk score, with which all LGG patients were divided into two groups including 65 low transcriptional risk score samples and 50 high transcriptional risk score samples.

Supplementary Figure 14 The validation of nomogram in CGGA dataset. **(A-C)**, the calibration curves of 1-, 3- and 5-years showed more appropriate calibration ability in CGGA cohort, which the blue dotted lines represented the ideal predictive model, and the red solid line represented the nomogram model. **(D)**, the time dependent ROC curves analysis for nomogram during 1-, 3- and 5-years in CGGA cohort, respectively.
