# Supplementary material for: A Novel Multi-Omics Analysis Model for Diagnosis and Survival Prediction of Lower-Grade Glioma Patients
Source: Front Oncol. 2022 May 12;12:729002. doi: 10.3389/fonc.2022.729002 (PMC9133344; doi:10.3389/fonc.2022.729002)
Supplement: Supplementary Figure 1 — The GO annotation and KEGG signaling pathway analysis in TCGA dataset. (A), the GO annotation exhibited several significant terms in IDHwt/1p19qnon-codel gliomas. (B), the KEGG signaling pathway demonstrated that multiple inflammation and tumor progress-related signaling pathways were significantly enriched in IDHwt/1p19qnon-codel gliomas. The GO annotation was performed by DAVID. The KEGG signaling pathway analysis was performed by ConsensusPathDB. [file DataSheet_1.zip › Supplementary Method.docx]

Supplementary Methods

**1. Sequencing data collection and preprocessing, identification of differentially expressed genes between isocitrate dehydrogenase (IDH) mutation and 1p/19q combined deletion (IDH^mut^/1p19q^codel^) and IDH wildtype and intact 1p/19q (IDH^wt^/1p19q^non-codel^) samples in The Cancer Genome Atlas (TCGA) dataset**

Firstly, RNA sequencing data and corresponding clinical and phenotype information of lower grade glioma (LGG) samples were extracted from the TCGA data portal (https://portal.gdc.cancer.gov/) using the NIH Genomic Data Commons (GDC). The incomplete data were eliminated and the remaining samples were grouped based on the status of IDH and 1p/19q (IDH^mut^/1p19q^codel^ vs IDH^wt^/1p19q^non-codel^). Gene expression profiles data were preprocessed by background correction, gene symbol transformation and normalization using RStudio (vision 4.0.0) and ActivePerl (version 5.28) software.

Secondly, the *limma* package (1) was utilized for screening the differentially expressed genes (DEGs) between 165 IDH^mut^/1p19q^codel^ samples and 94 IDH^wt^/1p19q^non-code^l samples from the TCGA cohort (HTSeq-FPKM of TCGA-LGG transcriptome profiling with complete clinical information). An absolute log2-fold change (|logFC |) of > 2 and an adjusted *P* value of < 0.05 were set as cutoff criteria to identify DEGs.

**2. Identification of DNA methylation driven genes between IDH^mut^/1p19q^codel^ and IDH^wt^/1p19q^non-codel^ samples in TCGA dataset**

DNA methylation data of 259 LGG samples was furthered extracted from the TCGA data portal using GDC. The methylation level of each gene was represented using beta value ranged from 0 to 1 (unmethylated to totally methylated). Additionally, DNA methylation profiles were also preprocessed using RStudio software. Afterwards, gene expression data and DNA methylation data were integrated from the same TCGA barcode structure. The DNA methylation driven gene (DMDGs) were defined as a group of genes whose DNA methylation levels were negatively related to the mRNA expression levels by linear regression analysis. Simultaneously, a mixed model was constructed for DNA methylation level between IDH^mut^/1p19q^codel^ and IDH^wt^/1p19q^non-codel^ samples and then the differential methylated genes were calculated by utilizing the Wilcoxon rank-sum test method (2).

**3. Gene ontology analysis, Kyoto Encyclopedia of Genes and Genomes and Gene set enrichment analysis**

DMDGs were used to perform gene ontology (GO) annotation, Kyoto Encyclopedia of Genes and Genomes (KEGG) using the Database for Annotation, Visualization and Integrated Discovery online tool (DAVID, https://david.ncifcrf.gov/) and ConsensusPathDB (http://cpdb.molgen.mpg.de/). Results were visualized using *clusterprofiler* package (3). The expression profiles of LGG samples were ordered by the constructed risk score level to divide all the LGG samples into two groups (High risk and Low risk) by employing X-tile to determine appropriate cutoff value (4) and then uploaded into the Gene Set Enrichment Analysis (GSEA) software to elucidate the enriched KEGG pathways that were significantly enriched in High risk group with the number of permutations set at 1000 and visualized results using R programming. False discovery rate (FDR) less than 0.05 and an absolute value of the enrichment score (ES) greater than 0.5 were defined as the cutoff criteria.

**4. Screening DNA methylation driven differentially expressed genes, development and validation of the predictive transcriptional risk score in TCGA dataset**

Firstly, DMDGs and DEGs were interacted to define DNA methylation driven differentially expressed genes (DME genes) which were hypomethylation/up-regulation and hypermethylation/down-regulation. To further narrow the scope of the candidate DME genes, the least absolute shrinkage and selection operator (LASSO) strategy was performed to select the most appropriate predictive variables from the TCGA cohort after primary filtration. Afterwards, the fold change and the correlation of methylation with expression were calculated by t-test and Pearson coefficient. Kaplan-Meier (K-M) curves were further utilized to perform survival analysis for DME genes based on optimum cutoff value using X-tile. The transcriptional risk score was calculated according to verified candidates using linear combination of coefficient derived from the LASSO regression model (β) multiplied by corresponding mRNA level.

**5. Selection cytosine-phosphate-guanine (CpG) sites of screened genes, development and validation of the predictive CpG risk score in TCGA dataset**

To further contrast with transcriptional risk score, all of CpG sites methylation value of each DME gene were selected and preprocessed using *ChAMP* package (5). These CpG sites derived from the Illumina Human Methylation 450 platform (450K array). Subsequently, the CpG sites were filtered based on univariate Cox regression analysis with *P* value < 0.01. The CpG risk score was calculated according to primary filtered CpG sites using linear combination of coefficient derived from the LASSO regression model (β) multiplied by corresponding CpG methylation value. To determine the optimal risk score model, the transcriptional risk score and CpG risk score were accurately estimated by pec package and the time-dependent receiver operating characteristic (ROC) curve.

To explore the correlation between primary selected CpG sites and de-/methyltransferase, the Molecular Signatures Database (MSigDB) (https://www.gsea-msigdb.org/gsea/msigdb/) with GO_DEMETHYLATION (GO:0070988) and GO_METHYLATION (GO:0032259) were utilized to calculate Pearson coefficient, and an absolute correlation coefficient (| *r* |) of > 0.7 and an adjusted *P* value of < 0.05 were set as cutoff criteria to further filtrate de-/methyltransferase. The Sankey diagram was used to visualize the annotation. The protein-protein interaction (PPI) was presented with the help of GeneMANIA (http://genemania.org/) to indicate the intrinsic connection with de-/methyltransferase and screened DME genes.

**6. Clinical significance evaluation in TCGA dataset**

The clinical significance of the transcriptional risk score model of LGG patients was evaluated by *t*-test, analysis of variance (ANOVA) and ROC for histopathological subtype, treatment outcome and overall survival (OS).

**7. Clinical samples collection and** **immunohistochemistry**

All tissue samples and corresponding clinical data were obtained from patient samples with LGG which underwent craniotomy in the First Affiliated Hospital of Xi’an Jiaotong University. Incomplete and loss to follow-up samples were eliminated. Immunohistochemistry (IHC) was performed as previously described (6). Primary antibodies were purchased from Abcam (anti-*TNFRSF12A*: cat. no. ab109365, anti-*DDIT4L*: cat. no. ab223034, anti-*TGFB2*: cat. no. ab36495, anti-*OCIAD2*: cat. no. ab118565 and anti-*MEOX2*: cat. no. ab262916) and Santa Cruz Biotechnology (anti-*EMP3*: cat. no. sc-81797). Goat anti-rabbit IgG (cat. no. ab97051, Abcam) and goat anti-mouse IgG (cat. no. ab205719, Abcam) were used as secondary antibodies. LGG tissues used for tissue microarrays (TMA) were embedded in paraffin, cut into 4-μm sections followed by deparaffinized, rehydrated, and staining with primary antibodies at 4℃ overnight. Next, the samples were incubated with secondary antibodies, stained with DAB and counterstained with hematoxylin. Images were taken using the light microscope. Subsequently, samples were assessed by two independent experienced pathologists. The immunohistochemical score (IHS), comprising values between 1 and 12, originated from the percentage of positive cells (1, < 10%; 2, 11% to 50%; 3, 51% to 80%; and 4, greater than 81%) multiplied by the score of staining intensity (0, negative; 1, weak; 2, moderate; and 3, strong) (7).

**8. Quantitative RT-PCR analysis using clinical samples**

The quantitative reverse transcription PCR (qRT-PCR) were performed as previously described (6). Briefly, total RNA was extracted using RNeasy mini kits according to the manufacturer’s protocol, and the concentration of RNA was determined by Nanodrop 2000. cDNA was synthesized according to the standard protocols and qRT-PCR was performed. GAPDH was used as an internal control. Relative mRNA expressions were calculated by 2^‐ΔΔt^ method. The primer sequences are listed as below:

*DDIT4L*-forward: TGTGGCTATCACCCAGAGAG, *DDIT4L*-reverse: TGCTTTGATTTGGACAGACAGT;

*EMP3*-forward: CCTGAATCTCTGGTACGACTGC, *EMP3*-reverse: GCCATTCTCGCTGACATTACTG;

*OCIAD2*-forward: TGCTTGTCACCCAGGGACTA, *OCIAD2*-reverse: CCTCACAGGTAAGGAGGCAGT;

*MEOX2*-forward: GCACCCGTTCTCCCAATCC, *MEOX2*-reverse: TCCCGCGATTATGCAAGATGA;

*TGFB2*-forward: CAGCACACTCGATATGGACCA, *TGFB2*-reverse: CCTCGGGCTCAGGATAGTCT;

*TNFRSF12A*-forward: GTGTTGGGATTCGGCTTGGT, and *TNFRSF12A*-reverse: GTCCATGCACTTGTCGAGGTC;

*GAPDH*-forward: ACCCAGAAGACTGTGGATGG, *GAPDH*-reverse: TTCAGC TCAGGGATGACCTT.

**9. Radiomics images collection and preprocessing, screening of radiomics features, development and validation of the predictive radiomics risk score in TCGA dataset**

Firstly, the corresponding magnetic resonance (MR) post-contrast T1-weighted images of LGG samples were downloaded from TCIA radiology portal (https://public.cancerimagingarchive.net/nbia-search/) using NBIA Data Retriever and further were eliminated incomplete or degraded images.

The contrast-enhancing images in DICOM format were imported into 3D Slicer (version 4.10.2) (http://www.slicer.org) and tumor region of interest (ROI) were manually segmented by two experienced evaluators using Paint tools in Segment Editor model to construct a 3D image in the intracranial space. Subsequently, the radiomic features of 3D image were extracted by Radiomics model. In brief, radiomics features consisted of 18 first order features (1st Order), 14 shape features, 24 gray level co-occurrence matrix (GLCM) features, 14 gray level dependence matrix (GLDM) features, 16 gray level run length matrix (GLRLM) features, 16 gray level size zone matrix (GLSZM) features and 5 neighboring gray tone difference matrix (NGTDM) features and the detailed definition of these features were described in *pyradiomics* documentation (8).

All of features were performed to z-score normalization by scaling mean values to 0 and a standard deviation to 1 before analysis. The inter-reader agreement for the radiomics features was assessed using intra-class correlation coefficient (ICC). The ICC represented the consistency of features derived by two evaluators and features with ICC ≥ 0.80 were considered as excellent reliability, 0.80 > ICC ≥ 0.5 as moderate reliability, and ICC < 0.5 as poor reliability (9).

After collecting and preprocessing of MR images as previously described, 85 samples and 107 radiologic features were incorporated in this study. The radiomics risk score was calculated based on primary filtered radiological features using linear combination of coefficient derived from the LASSO regression model (β) multiplied by corresponding radiomics feature value. The radiomics model was accurately estimated by calibration and discrimination to testify whether the radiomics model can distinguish the distribution of transcriptional risk score.

**10. Establishment and assessment of the nomogram in TCGA dataset**

Multivariate Cox regression analysis was performed to evaluate significant independent risk/protective factors, from which the predictive nomogram was established. The performance of the nomogram was also demonstrated by the ability of calibration and discrimination. Meanwhile, the appropriateness of the current predictors involved in nomogram was tested. As a result, the Schoenfeld residuals, the Deviance residuals, the variance inflation factor (VIF), the interaction and the Martingale residuals combined the restricted cubic spline (RCS) test were performed to evaluate the equally proportional hazards assumption, outliers, collinearity, interaction impact and the linear relationship between continuous independent variables with the logit transformation value of the hazard, respectively. Subsequently, the decision curve analysis (DCA) was also performed to assess the clinical applicability of nomogram and a net benefit for diverse prediction model at different threshold probabilities by adding the benefits and minimizing the harms (10).

**11. Validation nomogram and radiomics risk score in Chinese Glioma Genome Atlas (CGGA) dataset and clinical dataset**

The RNA sequencing data from CGGA database (http://cgga.org.cn/) with IDH^mut^/1p19q^codel^ and IDH^wt^/1p19q^non-codel^ LGG samples were obtained and the nomogram was further validated in the CGGA cohort. Similarly, the corresponding MR post-contrast T1-weighted images of clinical samples were collected and preprocessed as previously described. Then, radiomics risk score was further evaluated using clinical dataset.

**12. Verification** **copy number alteration and mutation characteristics of screened genes**

cBioPortal database (http://www.cbioportal.org/) and COSMIC database (https://cancer.sanger.ac.uk/cosmic/) were used to verified that the transcription levels of DME genes were related to corresponding DNA methylation alterations rather than copy number alteration (CNA) or mutations.

**13. Statistical analysis**

The statistical analyses were performed with RStudio software (version 1.3.1093; https://rstudio.com/products/rstudio/). Statistical significance levels were determined by two-sided tests and *P* < 0.05 was defined as statistically significant. The Mann-Whitney U-test and *t*-test was used for comparing two groups of continuous variables analysis and the χ2 test for categorical variables analysis. The LASSO regression was used to select variables.

# 14. Reference

1. Ritchie ME, Phipson B, Wu D, et al. limma powers differential expression analyses for RNA-sequencing and microarray studies. Nucleic Acids Research. 2015;43(7).

2. Gevaert O. MethylMix: an R package for identifying DNA methylation-driven genes. Bioinformatics. 2015;31(11):1839-1841.

3. Yu GC, Wang LG, Han YY, et al. clusterProfiler: an R Package for Comparing Biological Themes Among Gene Clusters. Omics-a Journal of Integrative Biology. 2012;16(5):284-287.

4. Camp RL, Dolled-Filhart M, Rimm DL. X-tile: a new bio-informatics tool for biomarker assessment and outcome-based cut-point optimization. Clin Cancer Res. 2004;10(21):7252-7259.

5. Morris TJ, Butcher LM, Feber A, et al. ChAMP: 450k Chip Analysis Methylation Pipeline. Bioinformatics. 2014;30(3):428-430.

6. Alafate W, Zuo J, Deng Z, et al. Combined elevation of AURKB and UBE2C predicts severe outcomes and therapy resistance in glioma. Pathol Res Pract. 2019;215(10):152557.

7. Lupp A, Hunder A, Petrich A, et al. Reassessment of sst(5) somatostatin receptor expression in normal and neoplastic human tissues using the novel rabbit monoclonal antibody UMB-4. Neuroendocrinology. 2011;94(3):255-264.

8. van Griethuysen JJM, Fedorov A, Parmar C, et al. Computational Radiomics System to Decode the Radiographic Phenotype. Cancer Res. 2017;77(21):e104-e107.

9. Lee M, Woo B, Kuo MD, et al. Quality of Radiomic Features in Glioblastoma Multiforme: Impact of Semi-Automated Tumor Segmentation Software. Korean J Radiol. 2017;18(3):498-509.

10. Van Calster B, Wynants L, Verbeek JFM, et al. Reporting and Interpreting Decision Curve Analysis: A Guide for Investigators. Eur Urol. 2018;74(6):796-804.
