# Supplementary material for: A Novel Multi-Omics Analysis Model for Diagnosis and Survival Prediction of Lower-Grade Glioma Patients
Source: Front Oncol. 2022 May 12;12:729002. doi: 10.3389/fonc.2022.729002 (PMC9133344; doi:10.3389/fonc.2022.729002)
Supplement: Supplementary Figure 1 — The GO annotation and KEGG signaling pathway analysis in TCGA dataset. (A), the GO annotation exhibited several significant terms in IDHwt/1p19qnon-codel gliomas. (B), the KEGG signaling pathway demonstrated that multiple inflammation and tumor progress-related signaling pathways were significantly enriched in IDHwt/1p19qnon-codel gliomas. The GO annotation was performed by DAVID. The KEGG signaling pathway analysis was performed by ConsensusPathDB. [file DataSheet_1.zip › Table S8.docx]

Table S8. The different time points of AUCs in two models.

| **Time** | **AUC (3-CpG)** | **AUC (6-Gene)** | ***P*** |
| --- | --- | --- | --- |
| 365 | 0.867 | 0.851 | 0.852 |
| 730 | 0.812 | 0.880 | 0.008 |
| 1095 | 0.824 | 0.894 | 0.046 |
| 1460 | 0.843 | 0.879 | 0.338 |
| 1825 | 0.855 | 0.887 | 0.423 |
| 2190 | 0.855 | 0.881 | 0.443 |
| 2555 | 0.785 | 0.807 | 0.549 |
| 2920 | 0.738 | 0.795 | 0.283 |

AUC: area under curve
