# Supplementary material for: A Novel Multi-Omics Analysis Model for Diagnosis and Survival Prediction of Lower-Grade Glioma Patients
Source: Front Oncol. 2022 May 12;12:729002. doi: 10.3389/fonc.2022.729002 (PMC9133344; doi:10.3389/fonc.2022.729002)
Supplement: Supplementary Figure 1 — The GO annotation and KEGG signaling pathway analysis in TCGA dataset. (A), the GO annotation exhibited several significant terms in IDHwt/1p19qnon-codel gliomas. (B), the KEGG signaling pathway demonstrated that multiple inflammation and tumor progress-related signaling pathways were significantly enriched in IDHwt/1p19qnon-codel gliomas. The GO annotation was performed by DAVID. The KEGG signaling pathway analysis was performed by ConsensusPathDB. [file DataSheet_1.zip › Table S1.docx]

Table S1. The characteristics of baseline.

| Characteristic | IDH^mut^/1p19q^codel^ | IDH^wt^/1p19q^non-codel^ | *P* |
| --- | --- | --- | --- |
| Age, median (IQR), years | 45.00  (35.00-54.00) | 57.00  (45.00-62.00) | <0.001 |
| Overal survival, days | 706.00  (436.00-1218.50) | 491.00  (241.00-724.00) | <0.001 |
| Status |  |  | <0.001 |
| Alive | 144 (87.27%) | 44 (46.81%) |  |
| Dead | 21 (12.73%) | 50 (53.19%) |  |
| Gender |  |  | 0.542 |
| Female | 74 (44.85%) | 42 (44.68%) |  |
| Male | 91 (55.15%) | 52 (55.32%) |  |
| WHO Grade |  |  | <0.001 |
| G2 | 79 (47.88%) | 19 (20.21%) |  |
| G3 | 70 (42.42%) | 67 (71.28%) |  |
| NA^*^ | 16 (9.70%) | 8 (8.51) |  |
| Pathology |  |  | <0.001 |
| NO | 66 (40.00%) | 9 (9.57%) |  |
| AO | 58 (35.15%) | 11 (11.70%) |  |
| MG | 36 (21.82%) | 17 (18.09%) |  |
| NA | 2 (1.21%) | 9 (9.57%) |  |
| AA | 3 (1.82%) | 48 (51.06%) |  |

IQR, interquartile range. IDH, isocitrate dehydrogenase. 1p19q, the chromosome 1p and 19q. G2 and G3, WHO grade 2 and grade 3. AA, anaplastic astrocytoma. NA, not otherwise specified astrocytoma. MG, mixed glioma. AO, anaplastic oligodendroglioma. NO, not otherwise specified oligodendroglioma. HR, hazrd ratio. *missing value. *P* was derived from the Mann–Whitney U-test and χ2 test.
