# Supplementary material for: A Novel Multi-Omics Analysis Model for Diagnosis and Survival Prediction of Lower-Grade Glioma Patients
Source: Front Oncol. 2022 May 12;12:729002. doi: 10.3389/fonc.2022.729002 (PMC9133344; doi:10.3389/fonc.2022.729002)
Supplement: Supplementary Figure 1 — The GO annotation and KEGG signaling pathway analysis in TCGA dataset. (A), the GO annotation exhibited several significant terms in IDHwt/1p19qnon-codel gliomas. (B), the KEGG signaling pathway demonstrated that multiple inflammation and tumor progress-related signaling pathways were significantly enriched in IDHwt/1p19qnon-codel gliomas. The GO annotation was performed by DAVID. The KEGG signaling pathway analysis was performed by ConsensusPathDB. [file DataSheet_1.zip › Table S6.docx]

Table S6. The correlation between genes expression and CpG sites.

| Genes | CpG Sites | Correlation coefficient | *P* |
| --- | --- | --- | --- |
| *DDIT4L* expression | cg00360769 | −0.692 | 2.884e−38 |
|  | cg02989600 | −0.535 | 1.479e−20 |
|  | cg03132532 | −0.374 | 5.196e−10 |
|  | cg05319305 | −0.795 | 1.221e−57 |
|  | cg08663159 | −0.517 | 4.274e−19 |
|  | cg12566138 | −0.446 | 4.284e−14 |
|  | cg12829717 | −0.602 | 6.465e−27 |
|  | cg17512904 | −0.497 | 1.483e−17 |
|  | cg18603154 | −0.571 | 8.93e−24 |
|  | cg18714412 | −0.664 | 2.544e−34 |
|  | cg25071651 | −0.81 | 2.188e−61 |
|  | cg27062369 | −0.474 | 6.07e−16 |
| *EMP3* expression | cg01795122 | −0.85 | 1.451e−73 |
|  | cg02871614 | −0.775 | 3.366e−53 |
|  | cg03208951 | −0.887 | 3.815e−88 |
|  | cg06350544 | 0.188 | 2.34e−03 |
|  | cg07605143 | 0.394 | 4.714e−11 |
|  | cg10338830 | 0.171 | 5.681e−03 |
|  | cg13633756 | −0.62 | 6.641e−29 |
|  | cg17315219 | 0.351 | 6.538e−09 |
|  | cg18449135 | 0.003 | 9.656e−01 |
|  | cg19805953 | −0.686 | 1.975e−37 |
|  | cg23344780 | −0.87 | 8.877e−81 |
| *MEOX2* expression | cg00003994 | −0.568 | 1.492e−23 |
|  | cg00839579 | −0.695 | 9.489e−39 |
|  | cg01181350 | −0.403 | 1.599e−11 |
|  | cg02908900 | −0.496 | 1.877e−17 |
|  | cg02969141 | −0.561 | 7.226e−23 |
|  | cg03985727 | −0.608 | 1.264e−27 |
|  | cg05494014 | −0.456 | 1.072e−14 |
|  | cg06411724 | −0.476 | 4.955e−16 |
|  | cg06879518 | −0.396 | 3.667e−11 |
|  | cg07395354 | −0.636 | 8.313e−31 |
|  | cg13047892 | −0.35 | 7.109e−09 |
|  | cg16331920 | −0.362 | 1.901e−09 |
|  | cg17093212 | −0.371 | 6.787e−10 |
|  | cg17544952 | −0.462 | 4.406e−15 |
|  | cg18580296 | −0.534 | 1.756e−20 |
|  | cg25859468 | −0.469 | 1.402e−15 |
|  | cg26675326 | −0.416 | 3.088e−12 |
|  | cg27096779 | −0.468 | 1.809e−15 |
|  | cg27638126 | −0.642 | 1.9e−31 |
| *OCIAD2* expression | cg00332212 | −0.545 | 1.795e−21 |
|  | cg04961553 | −0.602 | 6.833e−27 |
|  | cg08942875 | −0.689 | 9.608e−38 |
|  | cg10465672 | −0.794 | 2.02e−57 |
|  | cg12209042 | 0.092 | 1.414e−01 |
|  | cg13841742 | −0.751 | 3.812e−48 |
|  | cg16511076 | 0.014 | 8.235e−01 |
|  | cg18085333 | −0.143 | 2.091e−02 |
|  | cg23545105 | −0.816 | 3.849e−63 |
|  | cg24308654 | −0.15 | 1.584e−02 |
|  | cg26134090 | −0.591 | 9.719e−26 |
|  | cg26731119 | −0.695 | 1.135e−38 |
| *TNFRSF12A* expression | cg00510447 | −0.775 | 3.685e−53 |
|  | cg26808293 | −0.714 | 1.161e−41 |
| *TGFB2* expression | cg01558923 | −0.839 | 6.107e−70 |
|  | cg06899755 | −0.794 | 1.986e−57 |
|  | cg07810039 | −0.83 | 4.146e−67 |
|  | cg08746138 | −0.283 | 3.641e−06 |
|  | cg09167119 | −0.138 | 2.583e−02 |
|  | cg10484211 | −0.761 | 3.153e−50 |
|  | cg11976166 | −0.794 | 2.01e−57 |
|  | cg12461345 | −0.115 | 6.515e−02 |
|  | cg13285637 | −0.754 | 9.453e−49 |
|  | cg16361301 | −0.162 | 8.826e−03 |
|  | cg16658719 | −0.039 | 5.366e−01 |
|  | cg16899280 | −0.415 | 3.28e−12 |
|  | cg16967578 | −0.745 | 3.731e−47 |
|  | cg17934824 | −0.809 | 3.099e−61 |
|  | cg18876728 | 0.004 | 9.523e−01 |
|  | cg20698667 | −0.792 | 4.111e−57 |
|  | cg20991819 | −0.569 | 1.383e−23 |
|  | cg21387604 | −0.811 | 8.144e−62 |
|  | cg22021178 | −0.799 | 8.462e−59 |
|  | cg25132662 | −0.742 | 1.272e−46 |
|  | cg25851842 | 0.007 | 9.095e−01 |
|  | cg26343258 | −0.259 | 2.477e−05 |
|  | cg27508144 | −0.784 | 3.077e−55 |

CpG, cytosine-phosphate-guanine. *DDIT4L*, DNA Damage Inducible Transcript 4 Like, *EMP3*, Epithelial Membrane Protein 3, *MEOX2*, Mesenchyme Homeobox 2, *OCIAD2*, Ovarian Cancer Immunoreactive Antigen Domain Containing 2, *TGFB2*, Transforming Growth Factor Beta 2, *TNFRSF12A*, Tumor Necrosis Factor Receptor Superfamily Member 12A. *P* was derived from Pearson correlation test.
