# Supplementary figures and images for: A Novel Multi-Omics Analysis Model for Diagnosis and Survival Prediction of Lower-Grade Glioma Patients
Source: Front Oncol. 2022 May 12;12:729002. doi: 10.3389/fonc.2022.729002 (PMC9133344; doi:10.3389/fonc.2022.729002)

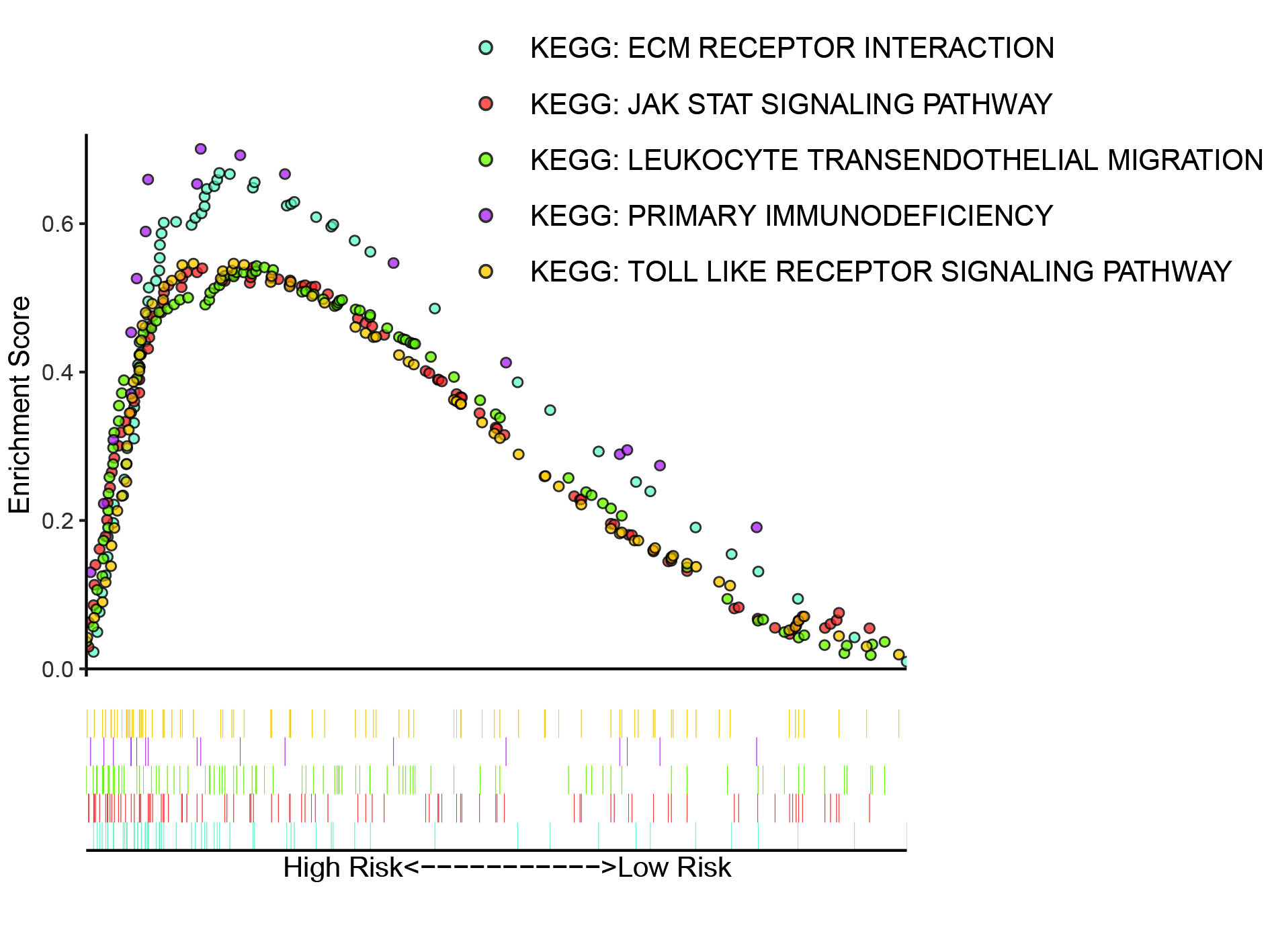

Supplement: Supplementary Figure 1 — The GO annotation and KEGG signaling pathway analysis in TCGA dataset. (A), the GO annotation exhibited several significant terms in IDHwt/1p19qnon-codel gliomas. (B), the KEGG signaling pathway demonstrated that multiple inflammation and tumor progress-related signaling pathways were significantly enriched in IDHwt/1p19qnon-codel gliomas. The GO annotation was performed by DAVID. The KEGG signaling pathway analysis was performed by ConsensusPathDB. [file DataSheet_1.zip › Figure S3.tif]

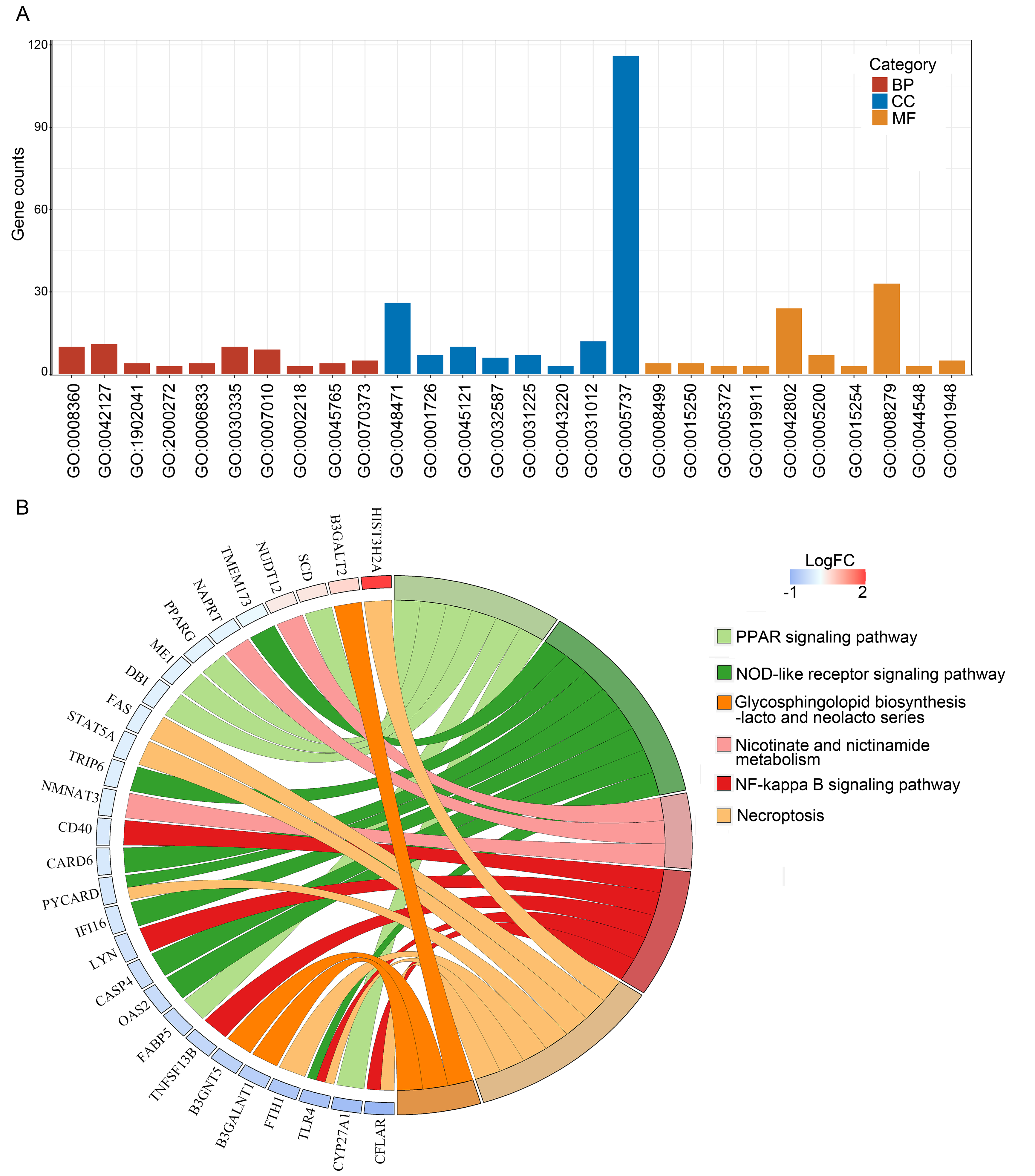

Supplement: Supplementary Figure 1 — The GO annotation and KEGG signaling pathway analysis in TCGA dataset. (A), the GO annotation exhibited several significant terms in IDHwt/1p19qnon-codel gliomas. (B), the KEGG signaling pathway demonstrated that multiple inflammation and tumor progress-related signaling pathways were significantly enriched in IDHwt/1p19qnon-codel gliomas. The GO annotation was performed by DAVID. The KEGG signaling pathway analysis was performed by ConsensusPathDB. [file DataSheet_1.zip › Figure S1.tif]

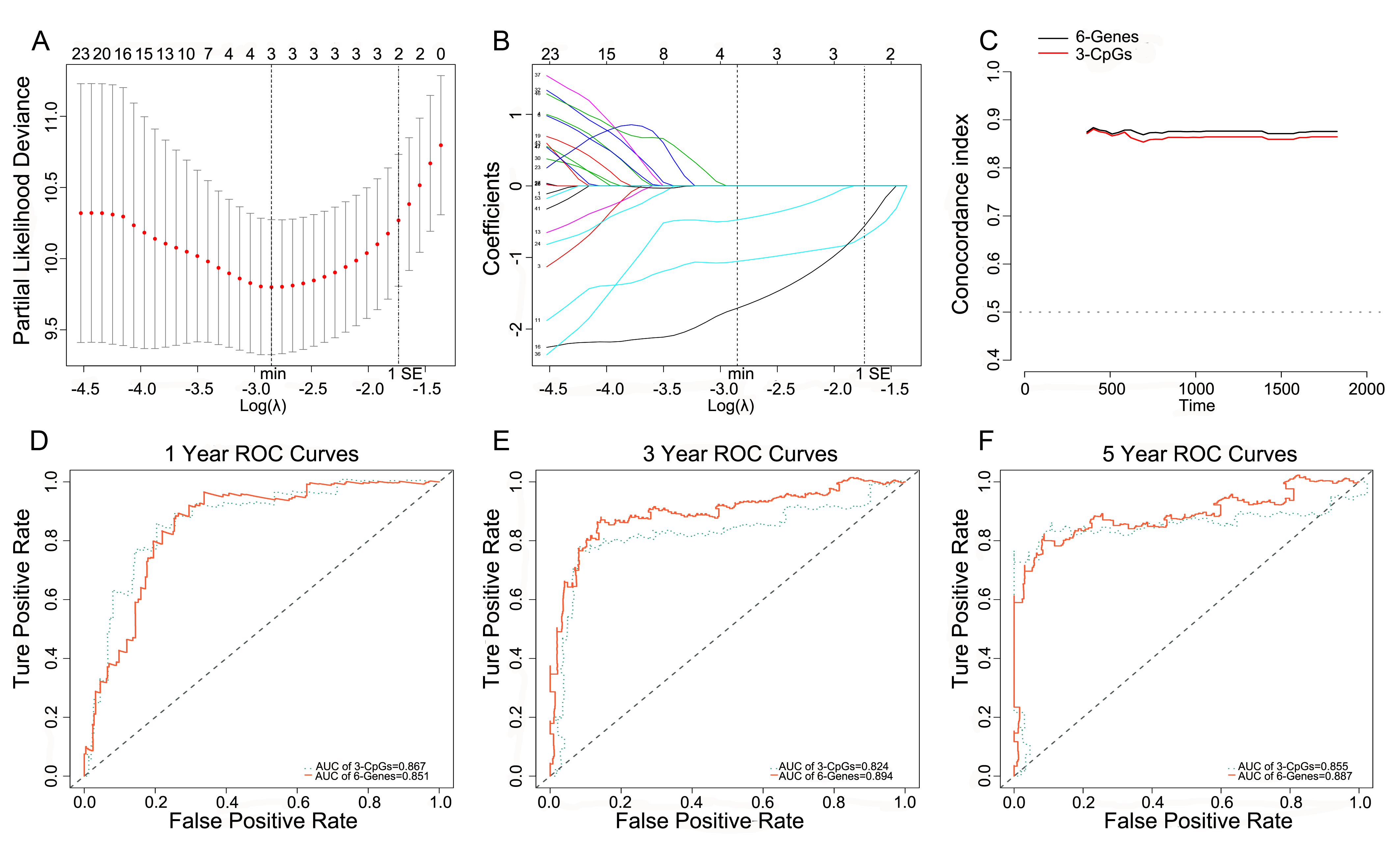

Supplement: Supplementary Figure 1 — The GO annotation and KEGG signaling pathway analysis in TCGA dataset. (A), the GO annotation exhibited several significant terms in IDHwt/1p19qnon-codel gliomas. (B), the KEGG signaling pathway demonstrated that multiple inflammation and tumor progress-related signaling pathways were significantly enriched in IDHwt/1p19qnon-codel gliomas. The GO annotation was performed by DAVID. The KEGG signaling pathway analysis was performed by ConsensusPathDB. [file DataSheet_1.zip › Figure S10.tif]

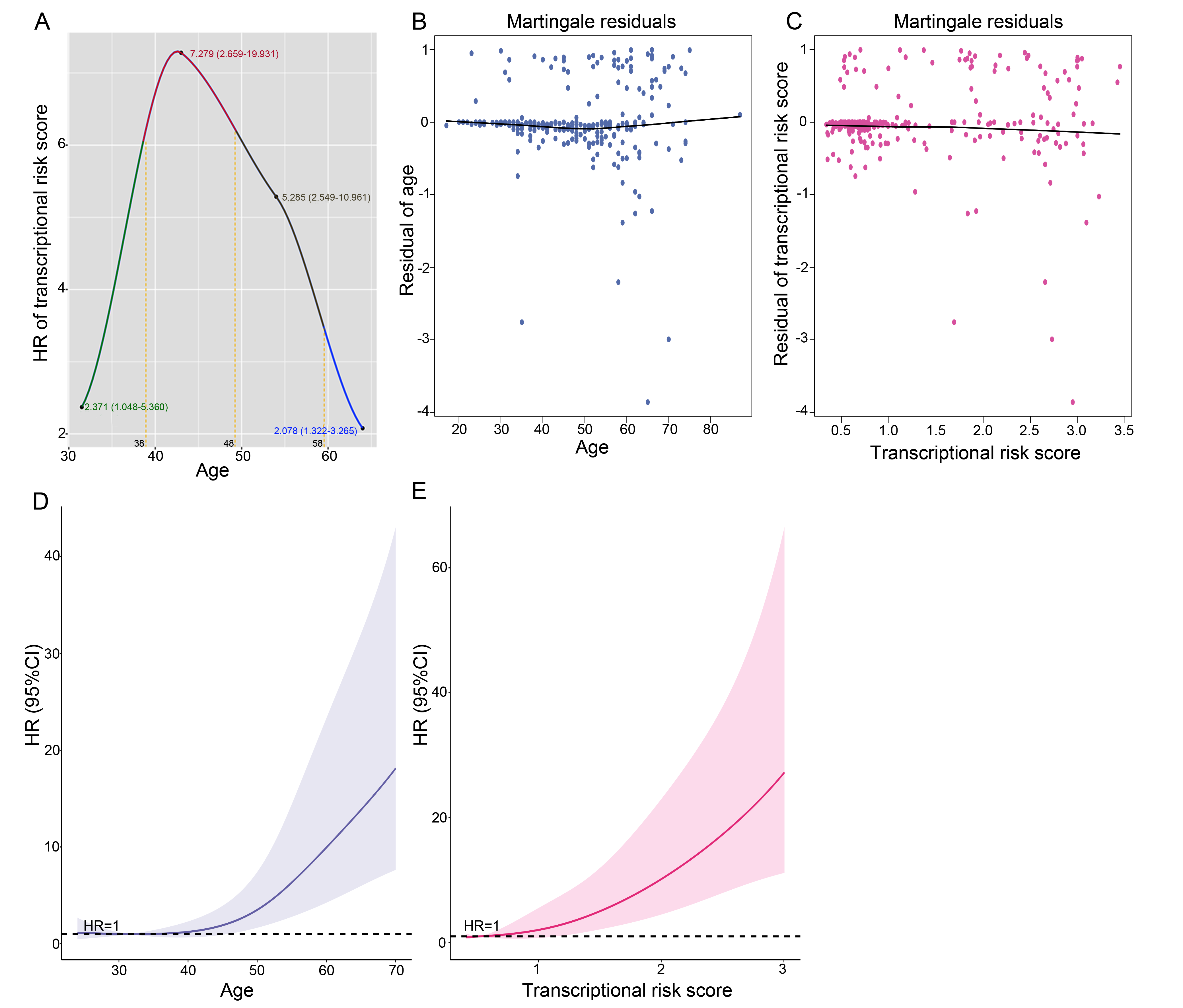

Supplement: Supplementary Figure 1 — The GO annotation and KEGG signaling pathway analysis in TCGA dataset. (A), the GO annotation exhibited several significant terms in IDHwt/1p19qnon-codel gliomas. (B), the KEGG signaling pathway demonstrated that multiple inflammation and tumor progress-related signaling pathways were significantly enriched in IDHwt/1p19qnon-codel gliomas. The GO annotation was performed by DAVID. The KEGG signaling pathway analysis was performed by ConsensusPathDB. [file DataSheet_1.zip › Figure S11.tif]

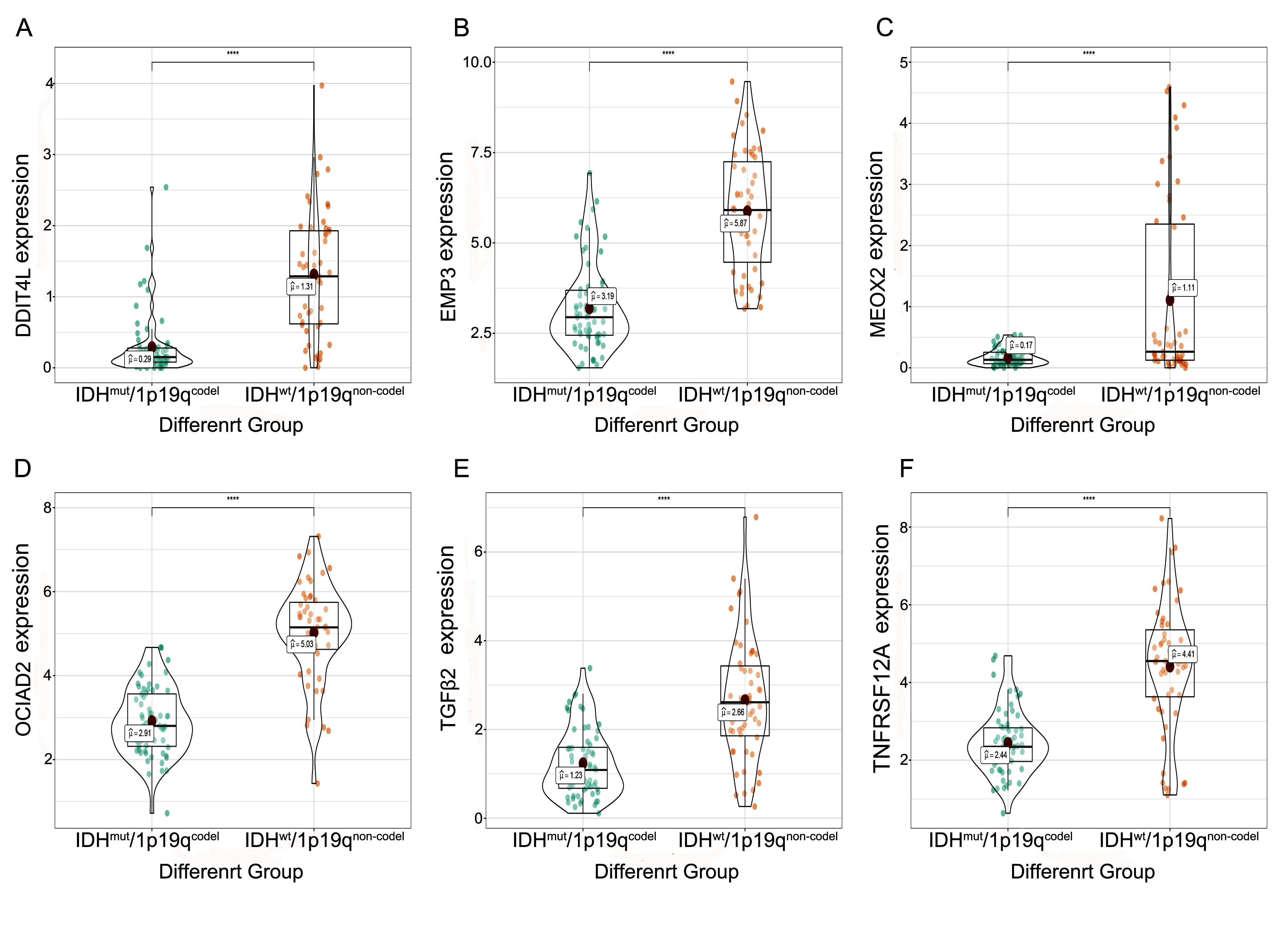

Supplement: Supplementary Figure 1 — The GO annotation and KEGG signaling pathway analysis in TCGA dataset. (A), the GO annotation exhibited several significant terms in IDHwt/1p19qnon-codel gliomas. (B), the KEGG signaling pathway demonstrated that multiple inflammation and tumor progress-related signaling pathways were significantly enriched in IDHwt/1p19qnon-codel gliomas. The GO annotation was performed by DAVID. The KEGG signaling pathway analysis was performed by ConsensusPathDB. [file DataSheet_1.zip › Figure S12.tif]

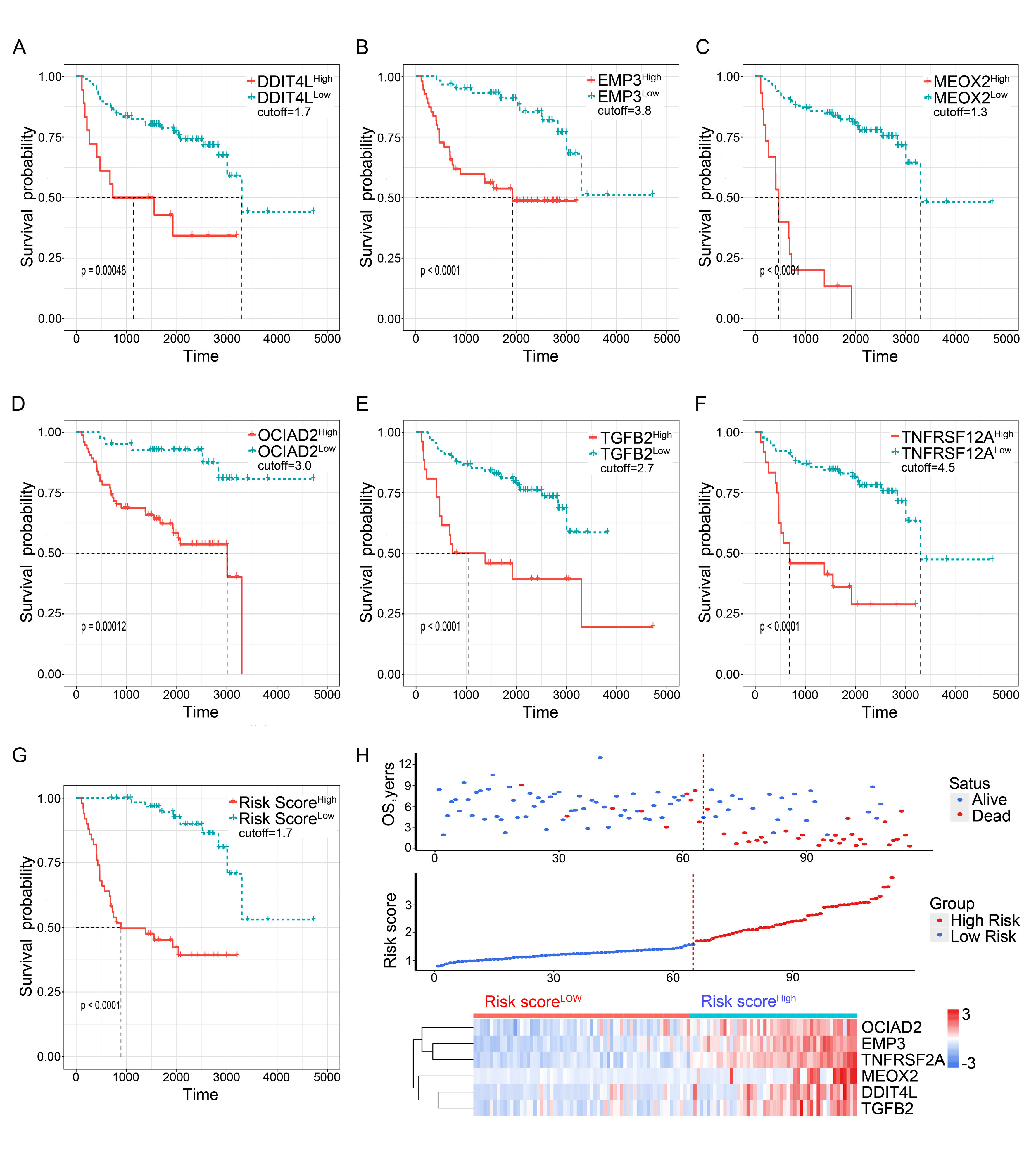

Supplement: Supplementary Figure 1 — The GO annotation and KEGG signaling pathway analysis in TCGA dataset. (A), the GO annotation exhibited several significant terms in IDHwt/1p19qnon-codel gliomas. (B), the KEGG signaling pathway demonstrated that multiple inflammation and tumor progress-related signaling pathways were significantly enriched in IDHwt/1p19qnon-codel gliomas. The GO annotation was performed by DAVID. The KEGG signaling pathway analysis was performed by ConsensusPathDB. [file DataSheet_1.zip › Figure S13.tif]

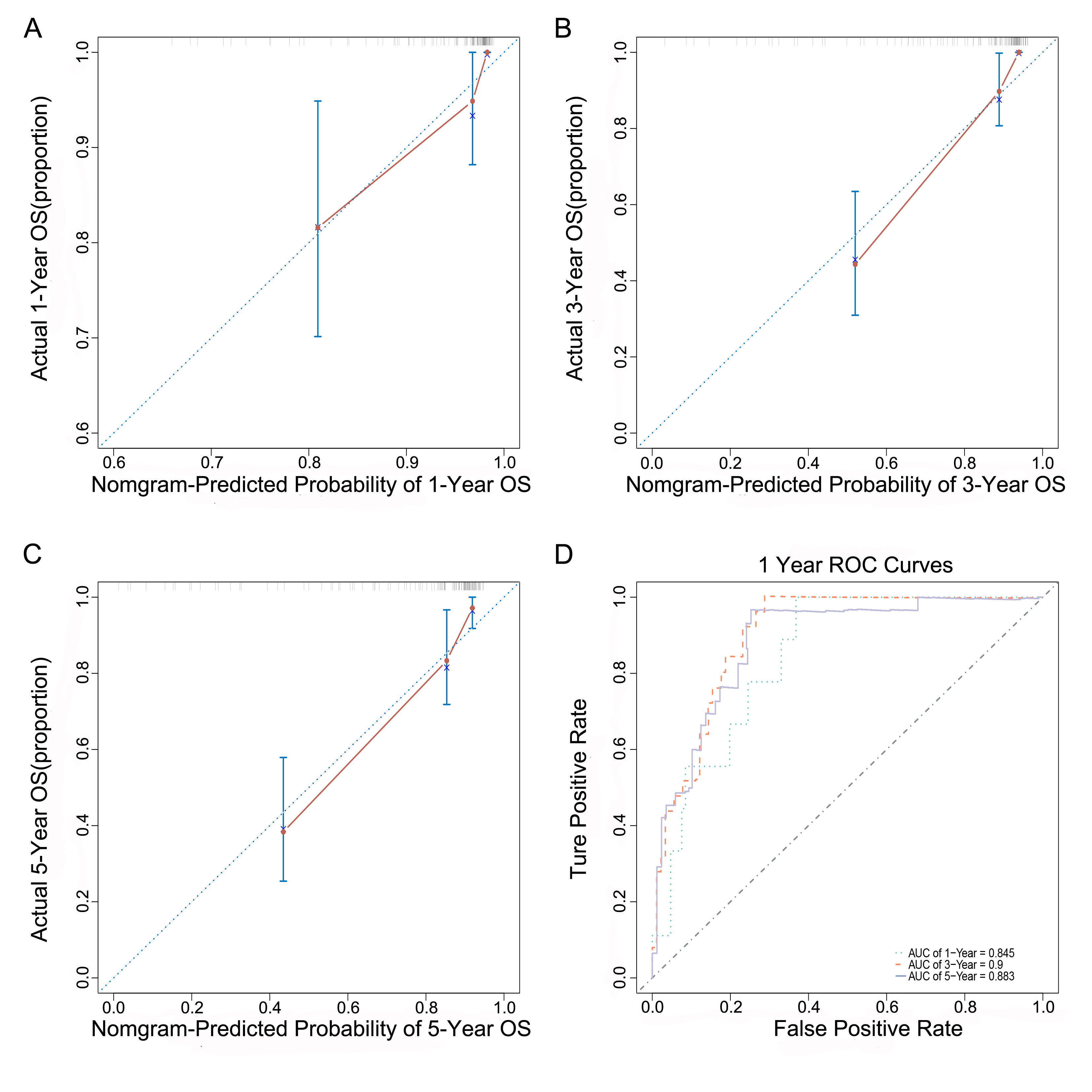

Supplement: Supplementary Figure 1 — The GO annotation and KEGG signaling pathway analysis in TCGA dataset. (A), the GO annotation exhibited several significant terms in IDHwt/1p19qnon-codel gliomas. (B), the KEGG signaling pathway demonstrated that multiple inflammation and tumor progress-related signaling pathways were significantly enriched in IDHwt/1p19qnon-codel gliomas. The GO annotation was performed by DAVID. The KEGG signaling pathway analysis was performed by ConsensusPathDB. [file DataSheet_1.zip › Figure S14.tif]

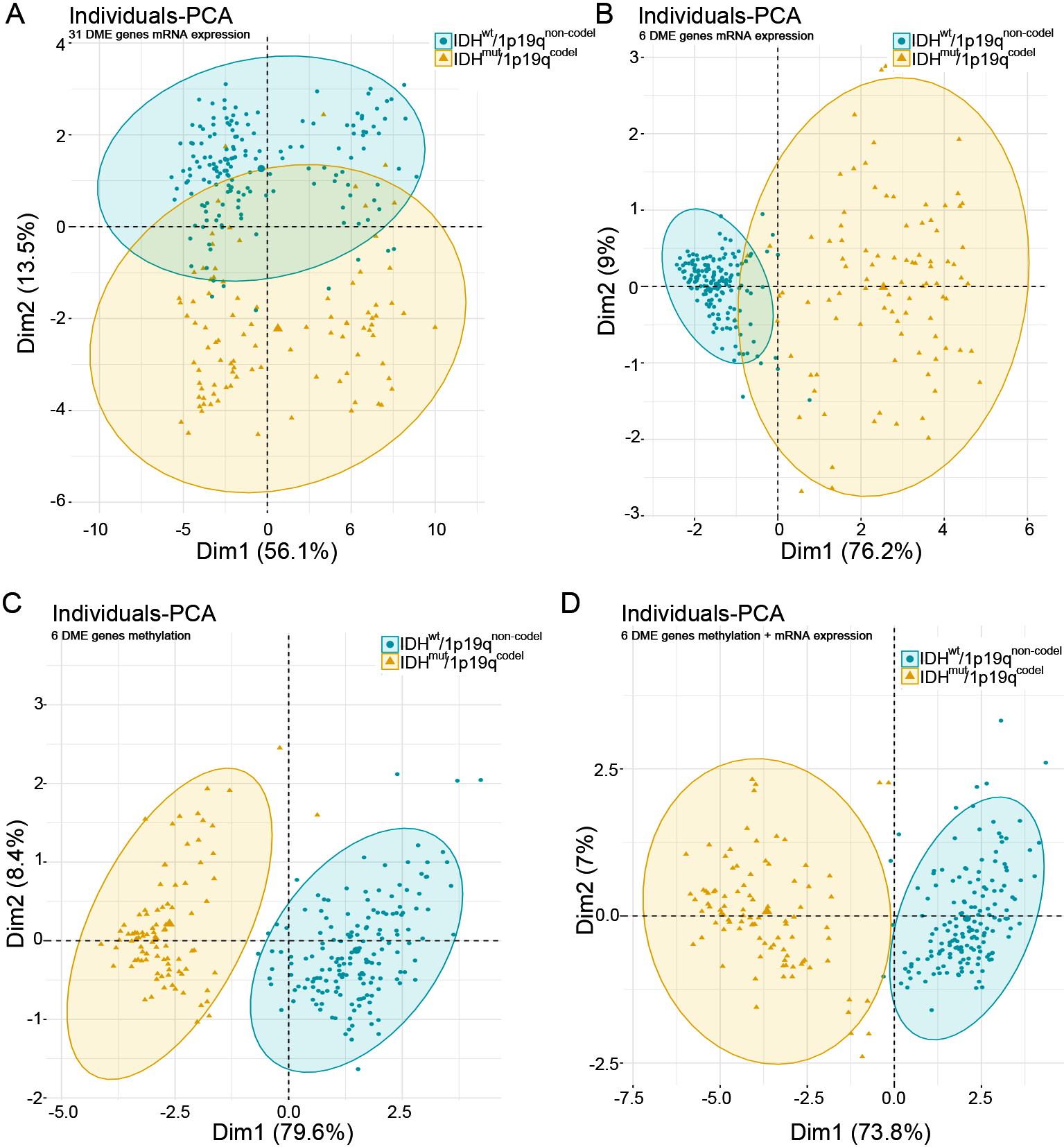

Supplement: Supplementary Figure 1 — The GO annotation and KEGG signaling pathway analysis in TCGA dataset. (A), the GO annotation exhibited several significant terms in IDHwt/1p19qnon-codel gliomas. (B), the KEGG signaling pathway demonstrated that multiple inflammation and tumor progress-related signaling pathways were significantly enriched in IDHwt/1p19qnon-codel gliomas. The GO annotation was performed by DAVID. The KEGG signaling pathway analysis was performed by ConsensusPathDB. [file DataSheet_1.zip › Figure S2.tif]

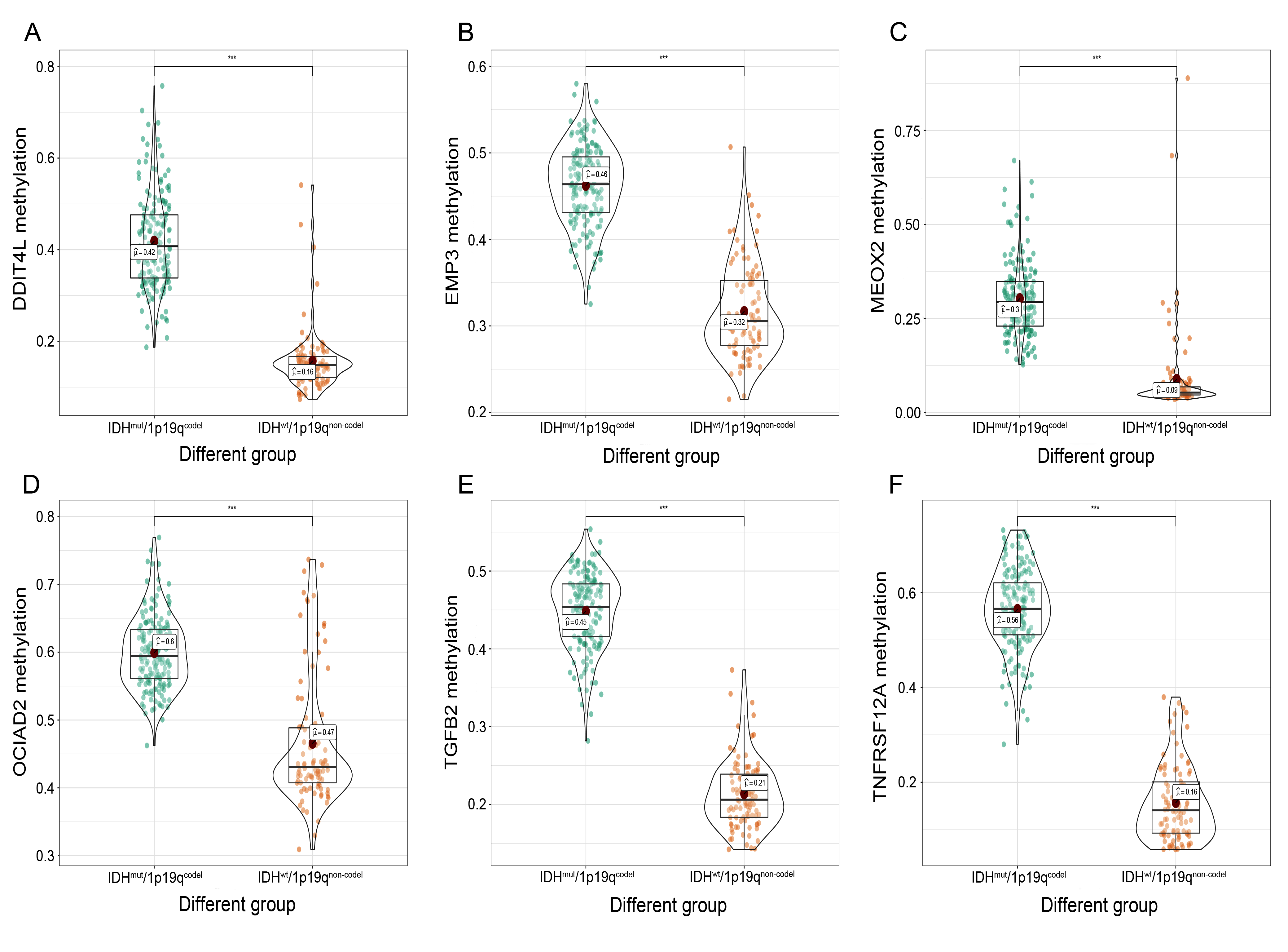

Supplement: Supplementary Figure 1 — The GO annotation and KEGG signaling pathway analysis in TCGA dataset. (A), the GO annotation exhibited several significant terms in IDHwt/1p19qnon-codel gliomas. (B), the KEGG signaling pathway demonstrated that multiple inflammation and tumor progress-related signaling pathways were significantly enriched in IDHwt/1p19qnon-codel gliomas. The GO annotation was performed by DAVID. The KEGG signaling pathway analysis was performed by ConsensusPathDB. [file DataSheet_1.zip › Figure S4.tif]

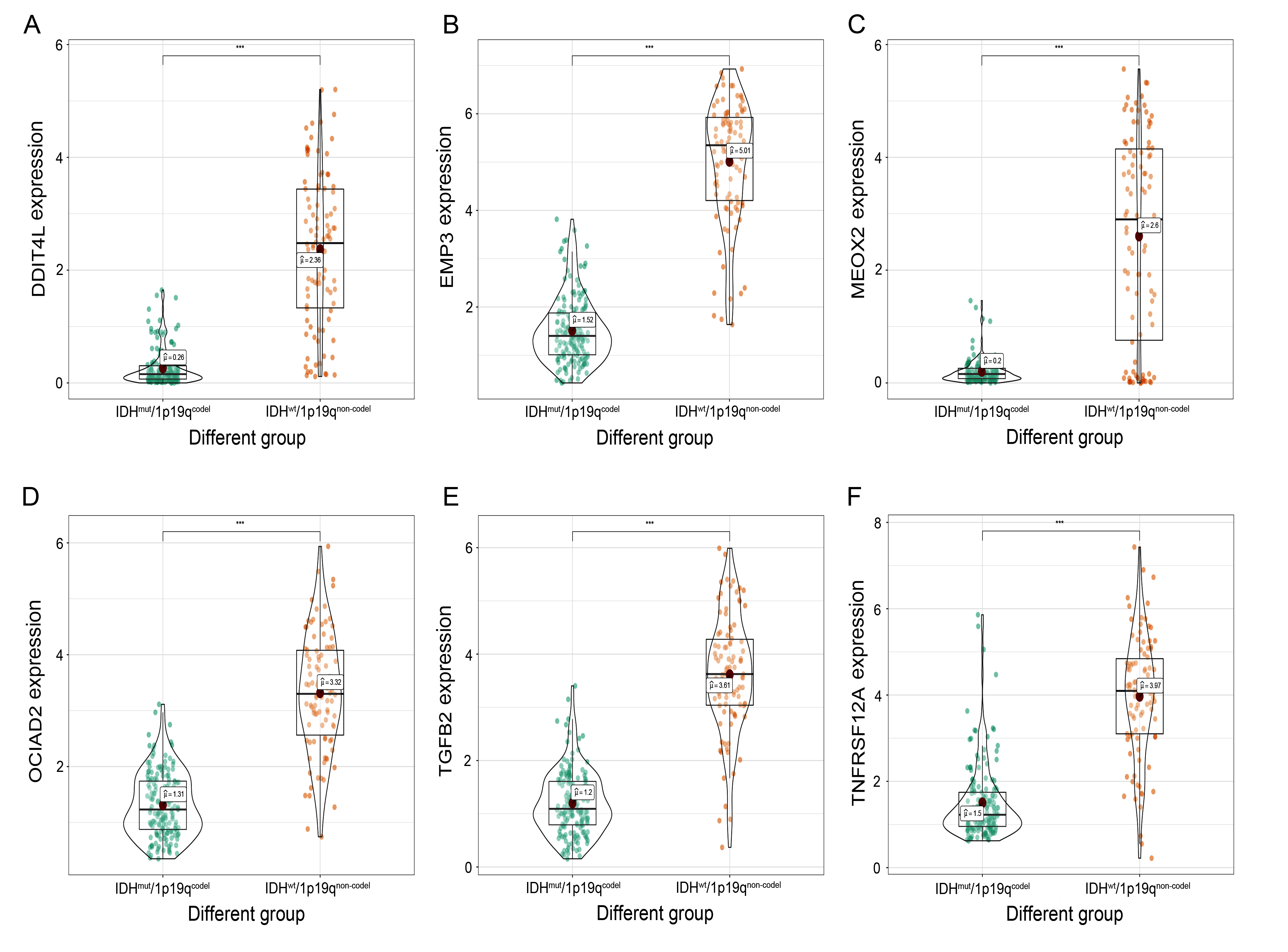

Supplement: Supplementary Figure 1 — The GO annotation and KEGG signaling pathway analysis in TCGA dataset. (A), the GO annotation exhibited several significant terms in IDHwt/1p19qnon-codel gliomas. (B), the KEGG signaling pathway demonstrated that multiple inflammation and tumor progress-related signaling pathways were significantly enriched in IDHwt/1p19qnon-codel gliomas. The GO annotation was performed by DAVID. The KEGG signaling pathway analysis was performed by ConsensusPathDB. [file DataSheet_1.zip › Figure S5.tif]

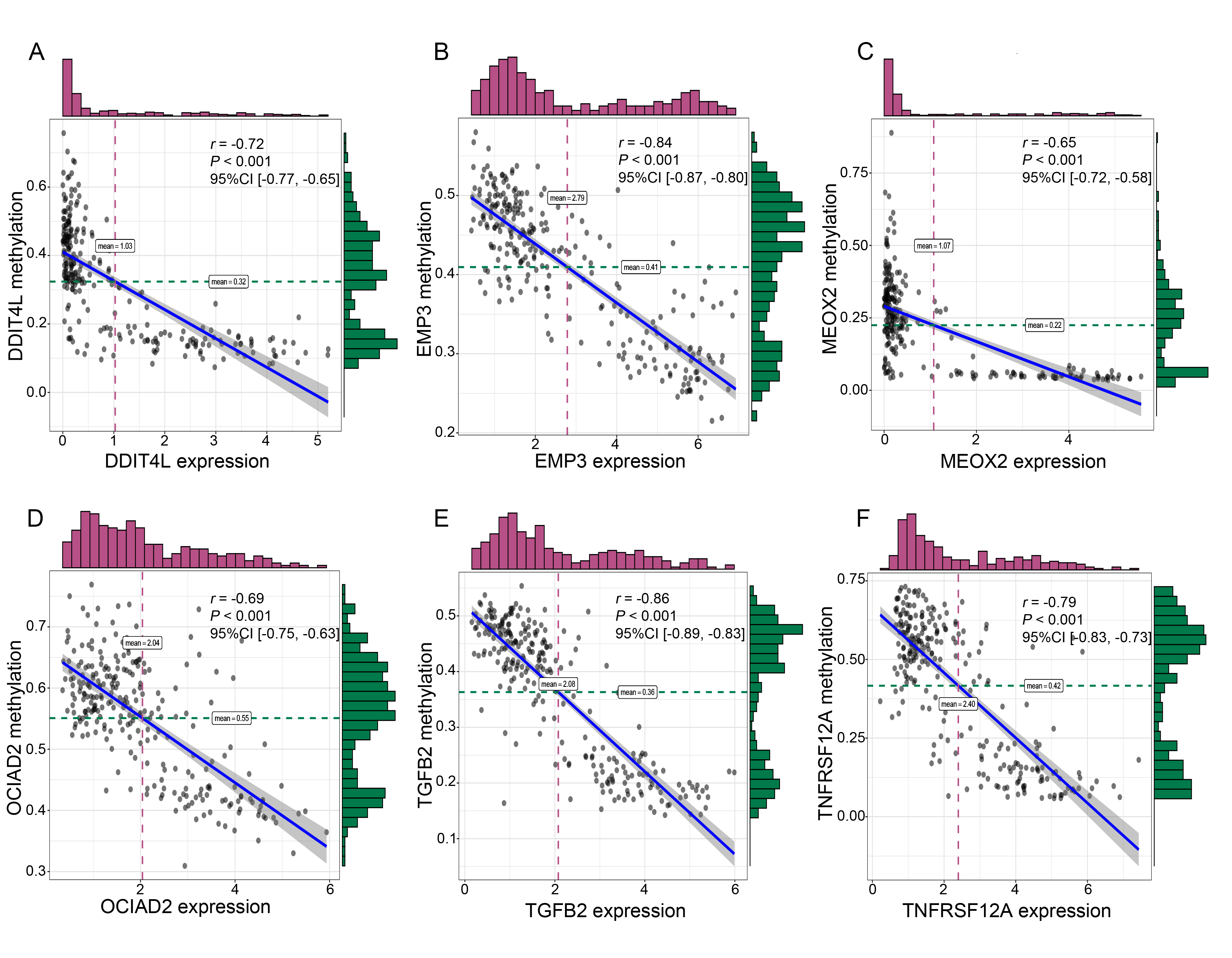

Supplement: Supplementary Figure 1 — The GO annotation and KEGG signaling pathway analysis in TCGA dataset. (A), the GO annotation exhibited several significant terms in IDHwt/1p19qnon-codel gliomas. (B), the KEGG signaling pathway demonstrated that multiple inflammation and tumor progress-related signaling pathways were significantly enriched in IDHwt/1p19qnon-codel gliomas. The GO annotation was performed by DAVID. The KEGG signaling pathway analysis was performed by ConsensusPathDB. [file DataSheet_1.zip › Figure S6.tif]

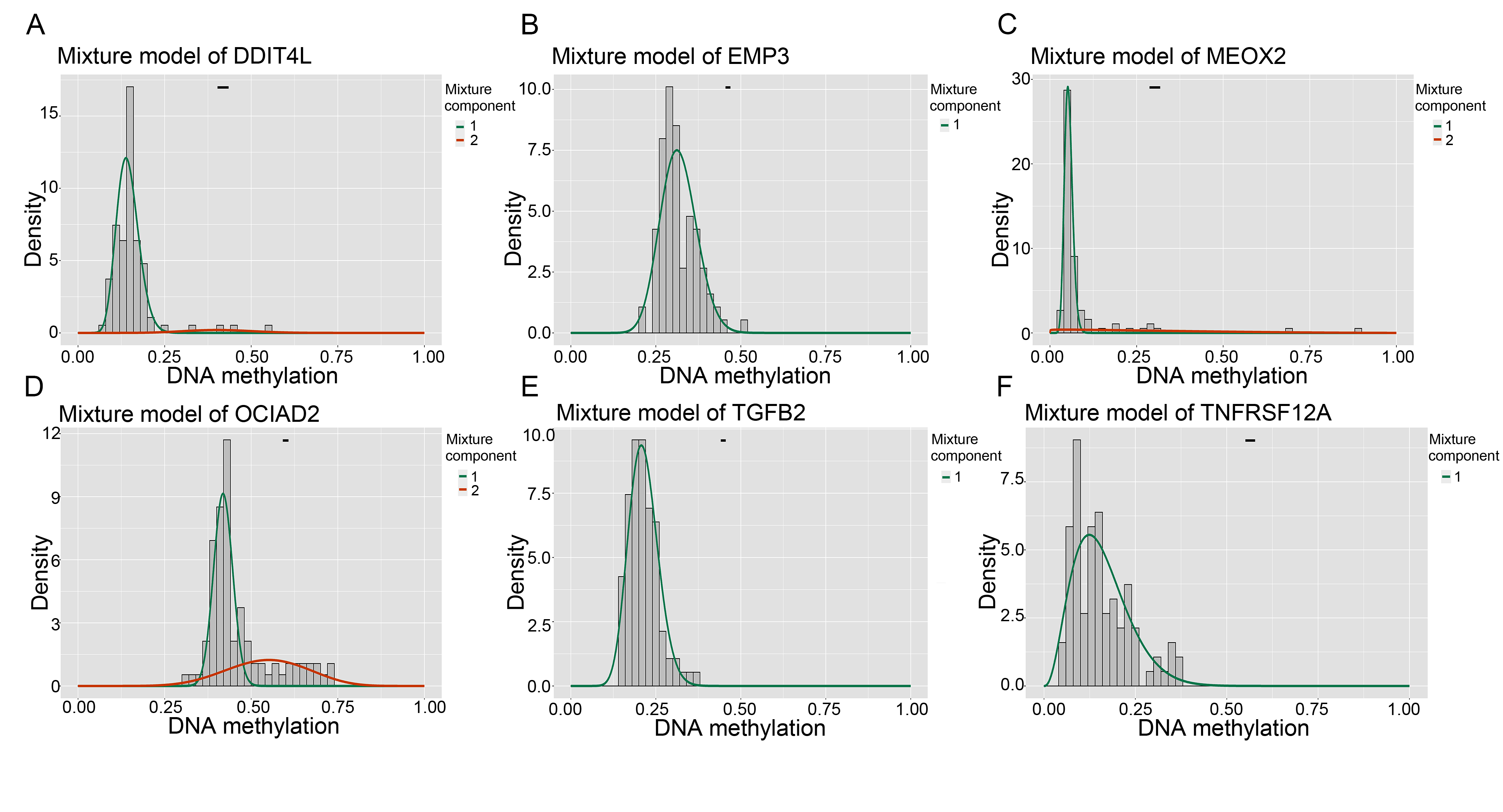

Supplement: Supplementary Figure 1 — The GO annotation and KEGG signaling pathway analysis in TCGA dataset. (A), the GO annotation exhibited several significant terms in IDHwt/1p19qnon-codel gliomas. (B), the KEGG signaling pathway demonstrated that multiple inflammation and tumor progress-related signaling pathways were significantly enriched in IDHwt/1p19qnon-codel gliomas. The GO annotation was performed by DAVID. The KEGG signaling pathway analysis was performed by ConsensusPathDB. [file DataSheet_1.zip › Figure S7.tif]

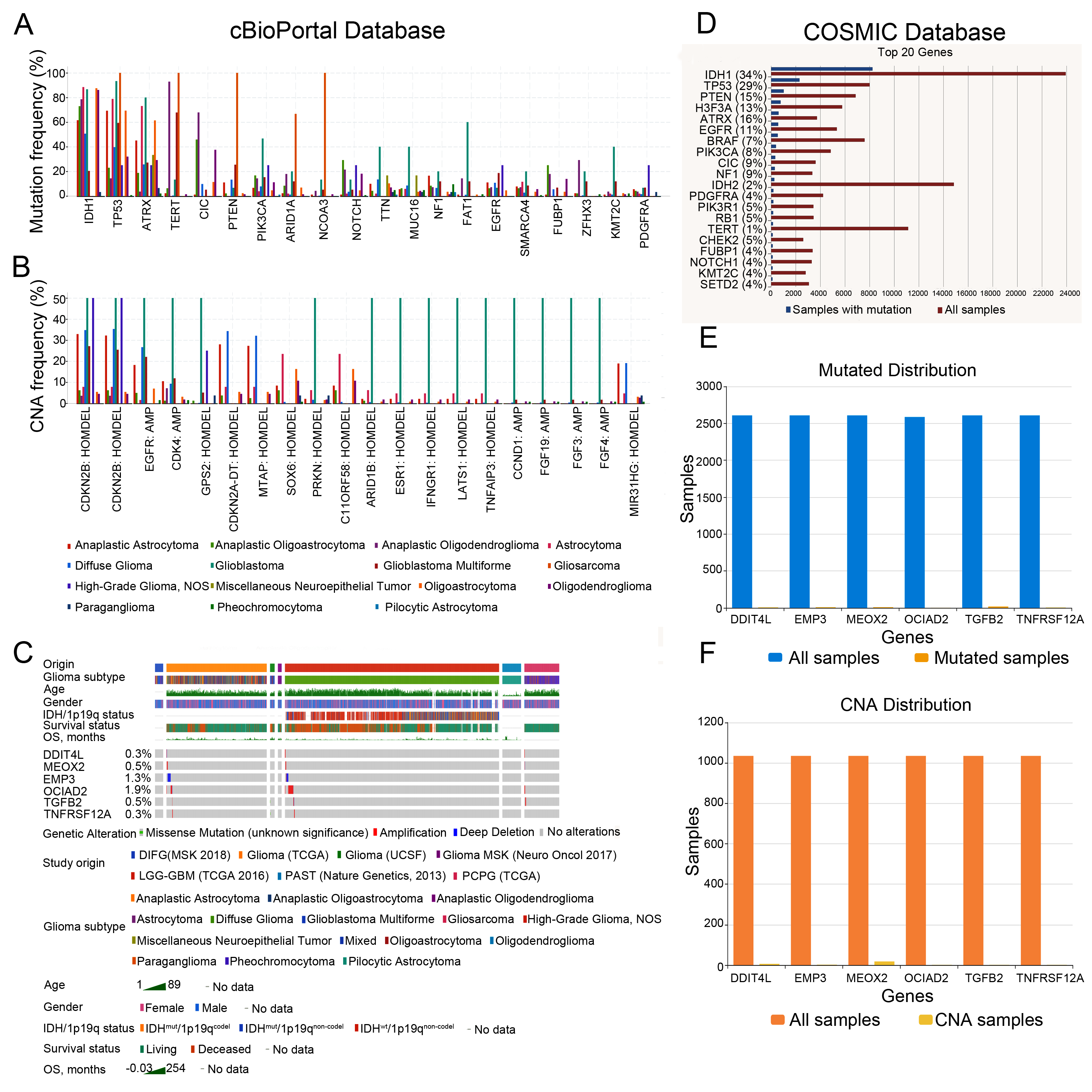

Supplement: Supplementary Figure 1 — The GO annotation and KEGG signaling pathway analysis in TCGA dataset. (A), the GO annotation exhibited several significant terms in IDHwt/1p19qnon-codel gliomas. (B), the KEGG signaling pathway demonstrated that multiple inflammation and tumor progress-related signaling pathways were significantly enriched in IDHwt/1p19qnon-codel gliomas. The GO annotation was performed by DAVID. The KEGG signaling pathway analysis was performed by ConsensusPathDB. [file DataSheet_1.zip › Figure S8.tif]

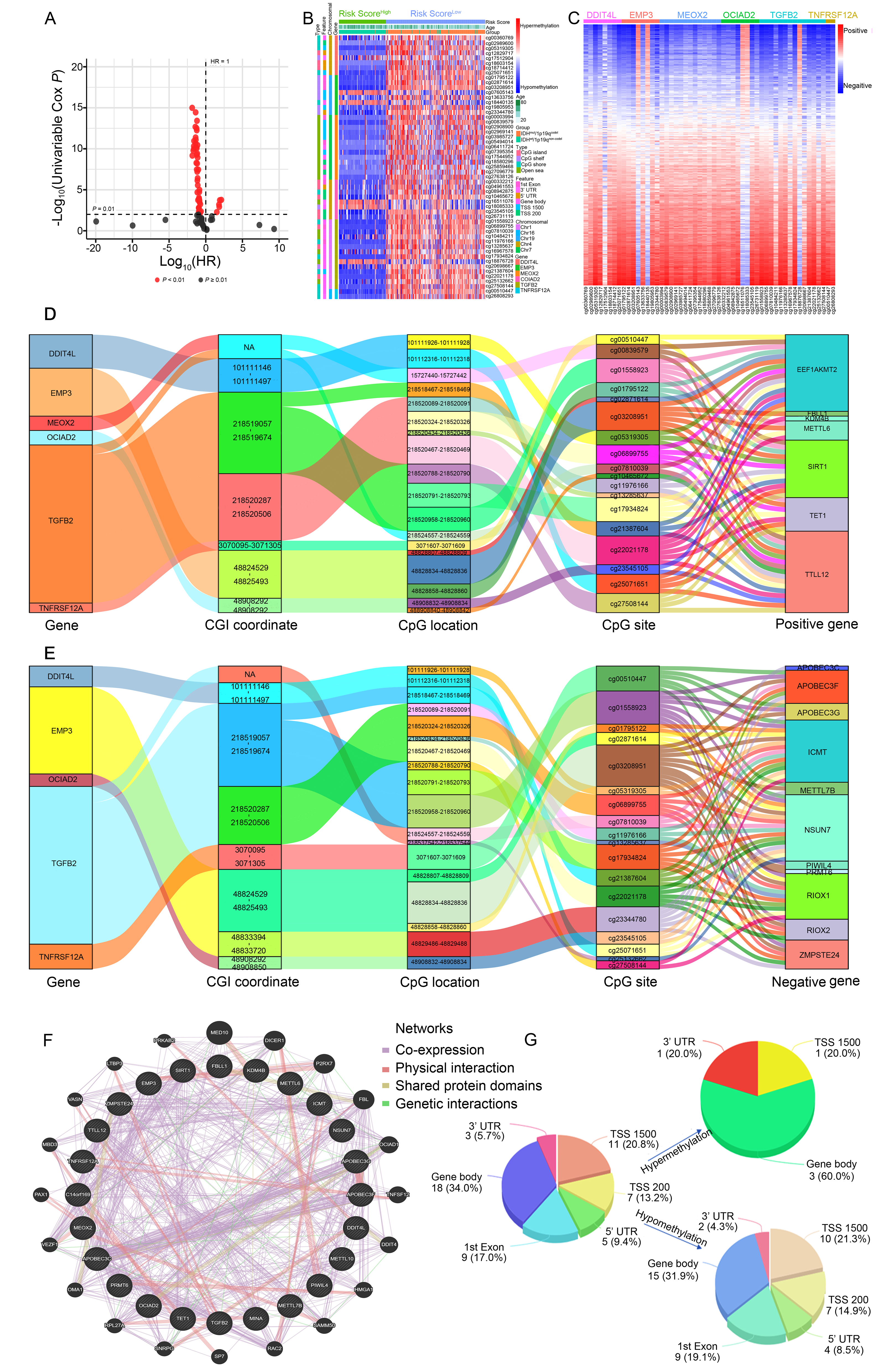

Supplement: Supplementary Figure 1 — The GO annotation and KEGG signaling pathway analysis in TCGA dataset. (A), the GO annotation exhibited several significant terms in IDHwt/1p19qnon-codel gliomas. (B), the KEGG signaling pathway demonstrated that multiple inflammation and tumor progress-related signaling pathways were significantly enriched in IDHwt/1p19qnon-codel gliomas. The GO annotation was performed by DAVID. The KEGG signaling pathway analysis was performed by ConsensusPathDB. [file DataSheet_1.zip › Figure S9.tif]
